# Managing admission and discharge processes in intensive care units

**DOI:** 10.1007/s10729-021-09560-6

**Published:** 2021-06-10

**Authors:** Jie Bai, Andreas Fügener, Jochen Gönsch, Jens O. Brunner, Manfred Blobner

**Affiliations:** 1 Department of Anesthesiology and Intensive Care Medicine, School of Medicine, University of Ulm, Albert-Einstein-Allee 29, 89081 Ulm, Germany; 2 Faculty of Management, Economics and Social Sciences, University of Cologne, Albertus-Magnus-Platz, 50923 Cologne, Germany; 3 Mercator School of Management, University of Duisburg-Essen, Lotharstraße 65, 47057 Duisburg, Germany; 4 Faculty of Business and Economics, University of Augsburg, Universitätsstraße 16, 86159 Augsburg, Germany; 5 Clinics for Anaesthesiology, Technical University of Munich, Klinikum Rechts der Isar, Ismaningerstraße 22, 81675 Munich, Germany

**Keywords:** Intensive care unit, Admission and discharge decisions, Markov decision process, Dynamic programming, Operations research

## Abstract

The intensive care unit (ICU) is one of the most crucial and expensive resources in a health care system. While high fixed costs usually lead to tight capacities, shortages have severe consequences. Thus, various challenging issues exist: When should an ICU admit or reject arriving patients in general? Should ICUs always be able to admit critical patients or rather focus on high utilization? On an operational level, both admission control of arriving patients and demand-driven early discharge of currently residing patients are decision variables and should be considered simultaneously. This paper discusses the trade-off between medical and monetary goals when managing intensive care units by modeling the problem as a Markov decision process. Intuitive, myopic rule mimicking decision-making in practice is applied as a benchmark. In a numerical study based on real-world data, we demonstrate that the medical results deteriorate dramatically when focusing on monetary goals only, and vice versa. Using our model, we illustrate the trade-off along an efficiency frontier that accounts for all combinations of medical and monetary goals. Coming from a solution that optimizes monetary costs, a significant reduction of expected mortality can be achieved at little additional monetary cost.

**Supplementary Information:**

The online version contains supplementary material available at 10.1007/s10729-021-09560-6.

## Highlights

- We model the decision-making process in the ICU and determine the optimal policy when a capacity shortage happens.
- The policies suggest direct implications for ICU management, such as reserving a certain number of beds for internal emergencies, or diverting ambulances if a certain threshold of critical patients is currently in the ICU.
- We discuss the trade-off between medical and monetary goals and evaluate an efficiency frontier for both objectives.

## Introduction

The intensive care unit (ICU) is one of the most crucial and expensive resources in the health care system [1]. Specialized equipment and highly skilled staff provide special care to the most severe and acute patients, leading to significant costs. In the US, costs for intensive care represent about 16.9%–38.4% of total hospital cost, which amounts to 5.2%–11.2% of national health expenditures [2, 3]. In order to cut costs, hospitals have aggressively reduced ICU beds [4]. As a consequence, the demand exceeds the capacity on a regular basis. Limited resources and increasing demand lead to overcrowding in many ICUs. As a result of this, Boyd and Evans [5] expect a shortfall of intensivist hours in the United States of 22% by 2020, and that this shortfall will increase to 35% by 2030.

ICU processes contain various uncertainties, which increase the difficulty of ICU management [6]. For example, the patient arrival pattern is hard to predict. Patients may be directly admitted to the ICU, arrive spontaneously after problems during a scheduled surgery, or transfer from the emergency department (ED), if necessary with a stopover in the operating room [7]. Among the patients in the ICU, the degree and severity of the disease as well as its subsequent treatment vary significantly. Furthermore, these health conditions will change during the stay in the ICU rapidly and unexpectedly. Thus, the length of stay (LOS) of an individual patient is hard to predict [8].

Patients in need of ICU beds are critically ill by definition. Most patients’ life-threatening conditions have to be treated immediately because delayed ICU admission is associated with higher mortality and additional resource expenditure ([9–11]). In this case, we don’t consider waiting as assumed in current literature [12–14]. When an additional patient unexpectedly needs intensive care treatment in a hospital with a congested ICU, there are two options – both associated with a major loss of time until sufficient treatment can be initiated. First, the patient could be transferred to another department or even another hospital with available ICU capacity. Until then, the situation might lead to patients being treated in the ED [11]. Second, a patient currently staying in the ICU is discharged earlier than planned to make space for the new patient. KC and Terwiesch [15] suggested such practice when the system load is high. Early discharge, however, requires bridging strategies including respective facilities. Many ICUs, e.g., provide an intervention room to stabilize the patients’ conditions and bridge for a short time until the bed is made available. Another option aims at surgical ICU patients expanding their treatment within the operating theatre, e.g., in the operating or recovery room. Such bridging approaches, however, do not substantially resolve the congestion of the respective ICU [12–14]. Neither of these options is desirable, because the morbidity and mortality of patients might increase [15]. Furthermore, patient pathways connect the ICU to other units inside and outside the hospital [16]. Decisions made in the ICU also influence upstream and downstream departments [7]. Capacity shortages in the ICU can also cause congestion of the patient flow within the entire hospital, e.g., by blocking transferals from the ED. Additionally, overloaded staff and decreased revenues are other possible negative effects. Thus, making good admission and discharge decisions is crucial to managing ICU capacities efficiently and simultaneously ensuring a high service quality.

In many ICUs, including the case study hospital, a myopic strategy (that is, only considering direct and immediate effects) of patient admission and discharge control is applied: As long as free beds are available, any new arriving patient is admitted. In case of capacity shortages, different myopic policies (such as the early discharging of existing patients or the rejection of the arriving patient) are applied to minimize the direct negative consequences that are typically evaluated based on the judgment of the ICU physicians. Strategies applied in practice are discussed in several papers [12, 17–19]. Although these myopic strategies are easy to implement, they have shortcomings. For example, when the last available bed is assigned to a patient who might also be diverted or delayed, the next arriving patient who cannot either be diverted or delayed will cause an issue. The American College of Critical Care Medicine defines and regularly updates guidelines on ICU admission, discharge, and triage decisions [20]. They identify the prioritization of patients and management of scarce ICU resources as an open issue: Instead of providing a clear recommendation, they conclude that “further research is needed on all aspects of rationing critical care resources to narrow the current gaps in allocating scarce resources”.

**Fig. 1:**
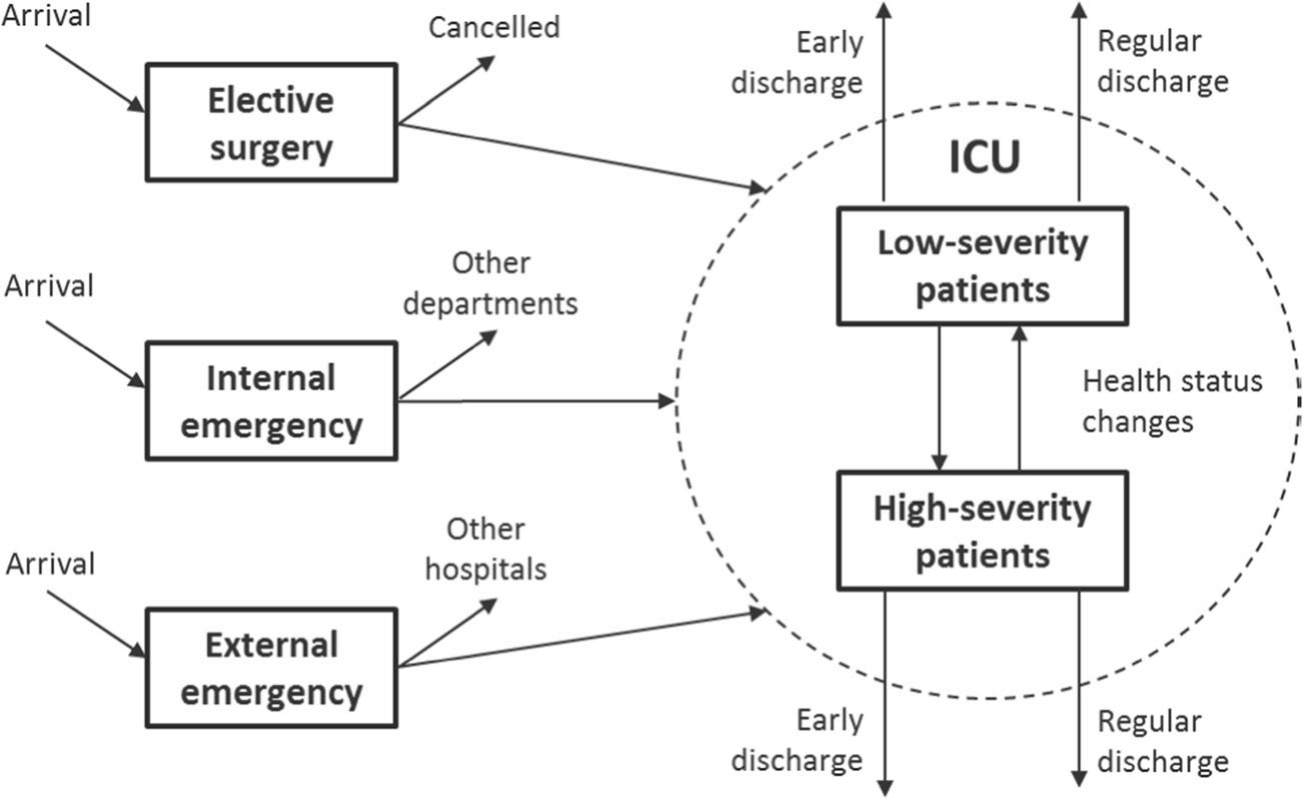
The patient flow in the ICU

To help answer these questions, we consider optimal ICU admission and discharge policies in an analytical model. It shows that capacity allocation and rationing issues are central and at the heart of important operational questions: When should an ICU admit or reject arriving patients? Should ICUs reserve capacity in order to be able to admit critical patients most of the time or rather focus on high utilization? Should an arriving patient be admitted, although this necessitates prematurely discharging another? Obviously, both admission control of arriving patients and demand-driven early discharge of currently residing patients are operational decisions and should be considered simultaneously. Naturally, when employing additional staff in the ICU, more patients can be treated. But the fixed cost of staffing will also be increased, and the training cost should be considered as well. Finally, the above-mentioned bridging approaches are an inherent precondition of such solutions. Actually, more staff and ad hoc available facilities can be assumed as additional ICU capacity. In our model, we assume that the capacity of the ICU (both beds and staffing irrespectively the labelling) is fixed. To do so, we use the stylized model of ICU admission and discharge visualized in Fig. 1. As in Litvak et al. [18], patient arrivals may be differentiated between three types: The first type includes patients following *elective surgeries*, where the nature of the surgery typically requires intensive care. The arrival times of these patients depend, prima facie, on the surgical schedule. However, the uncertainties in operating room scheduling [21] as well as in the surgery process [22] make the time of arrival at the ICU stochastic. The second type comprises *internal emergency patients*. These patients have already been admitted to the hospital, and unexpectedly require intensive care. Typical examples are routine surgical procedures which become more complex and lead to the patient now requiring intensive care, or readmissions following early discharges from the ICU. The final type of arriving patients describes *external emergency patients*, who are mostly brought in by ambulance.

To admit a surgical patient to the ICU, many complex criteria are needed to be considered, e.g., the preoperative health status, the invasiveness, the extent of surgery, the surgical organ, and the degree of tissue trauma. The prediction is complex and characterized by a considerable amount of uncertainty. This dilemma is reflected in the fact that 70% of all deaths after surgery in hospitals occur in normal wards rather than in ICUs [23]. Due to the increasing use of Big Data analyses in healthcare, machine learning algorithms have been superior to traditional prediction accuracy scores [24]. Recently, suggested a highly accurate postoperative risk prediction model for ICU admissions Jauk et al [25]. Even if advanced models accurately predict the probability of postoperative ICU demand on a personalized level, the exact prediction alone lacks operational benefit. The key question is whether a hospital provides for *n* scheduled patients with a predicted (even exact) individual ICU treatment probability *p*_*i*_ a total of *n* or *n* ∙ *p*_*i*_ beds. Since probabilities for ICU demand usually are right skewed distributed, the answer to this question is critical. Nevertheless, any request for postoperative ICU treatment is decided before elective surgery and, therefore, part of the scheduling process for major elective surgery. Unexpected cancelation of the reservation always leads to re-scheduling of surgery. Actually, the surgery-related factors cannot be estimated sufficiently before surgery is completed. They mainly contribute to the severity of ICU treatment, which therefore has to be estimated at admission to the ICU and need to be re-evaluated on each treatment day.

In the case of an internal emergency, an already hospitalized patient will be transferred to the ICU – in case of congestion, emergency care has to be provided in another department of the hospital. These patients’ medical history is given in the hospital chart. But it does not hold true for the current cause of the deterioration of the condition. Importantly, even during the recent COVID-19 pandemic, such patients are not transferred to other hospitals. Instead, other ICU patients in more stable conditions are transferred. External emergency patients with a request for ICU treatment are at their best discussed with the out-hospital emergency team resulting in working diagnoses and an estimate of the worst complication to be averted. Actually, their physical status is unknown before admission to the ICU. In the case of congestion, ambulances are diverted to other hospitals with available ICU capacity.

Consequently, almost every ICU patient’s health status is reliably determined at admission to the ICU but not earlier. Our model dichotomizes the grade of severity, which maps the current human-based decision algorithm best: high-severity and low-severity patients. High-severity patients are characterized by a more critical condition, going along with a longer expected LOS compared to low-severity patients. During their stay at the ICU, the health status of high-severity patients may improve to the low-severity status, and the conditions of low-severity patients might also worsen. Moreover, both types of patients are regularly discharged from the ICU; in case their condition further improves, they are transferred to another unit, or in case of death. Please note that the model can be extended straightforwardly to include more arrival types that enable a more differentiated advanced estimation of a patient’s health status by considering, for example, “safe” electives (e.g. hand surgery, young people) and “risky” electives (e.g. heart surgery, elderly), with different probabilities of the patient’s status being high-severity. The options of admission and discharge control are admitting or rejecting an arriving patient, and early discharging an existing patient (“early discharge”). Both rejection and early discharge result in negative effects to patients and hospitals, both from a medical and a monetary point of view.

In this paper, we employ a discrete time Markov decision process (MDP). This modeling approach is standard in comparable stochastic dynamic problems with subsequent, interdependent decision opportunities. The objective is to minimize the negative consequences of capacity shortages. Denied admissions and early discharges are penalized. We evaluate the policy resulting from the MDP in two case studies capturing different management objectives – a medical and a monetary perspective – based on real-world data from a large German teaching hospital. The results show that the optimal policy from our MDP model can considerably reduce the negative effects from a medical perspective – the mortality due to capacity shortages may be reduced by 21% in our case study compared to myopic policies. In contrast, myopic policies mimicking intuitive decisions seem to work well from a monetary perspective. However, both perspectives are not aligned and may lead to considerably different decisions and results. Focusing on monetary instead of medical goals, for instance, leads to an increase of expected mortality of nearly 50%. To illustrate the trade-off between both perspectives, we draw an efficiency frontier that includes a representative sample of combinations of medical and monetary goals. We discuss the impact of different combinations of cost parameters on solutions and on the robustness of our model in case of over- or underestimation of cost parameters.

Our approach provides a novel contribution in two directions: First, it enables an analytical demonstration of the trade-offs between medical and monetary goals when designing admission and discharge policies in ICUs. The impact of different goals is large, and deciding on the percentage of resources to be spent on intensive care is of great societal importance. Second, our model provides optimal holistic policies combining admission and discharge decisions in an ICU based on realistic assumptions. Those policies may lead to direct implications for ICU management, such as reserving a certain number of beds for internal emergencies, or diverting ambulances if a certain threshold of critical patients is currently in the ICU. The policies our stylized model produces are of a low complexity level, which means that they can be printed out and be directly used by ICU managers. Thus, there are no requirements on certain information systems that have to be in place in order to implement such policies in practice.

The remainder of the paper is organized as follows. After reviewing the literature on ICU admission and discharge problems in Section 2, we describe the problem and present the MDP model in Section 3. Section 4 explains the data for the case studies. Section 5 contains the results of the case studies. We describe the optimal policies of a medical and a monetary objective, analyze their performance, and briefly discuss strategic implications. We perform sensitivity analyses in Section 6, including an efficiency frontier discussion that looks at combinations of medical and monetary goals by considering 32 different scenarios with different combinations of cost parameters, and a study on the robustness of our model to over- and underestimation of cost parameters. Finally, Section 7 concludes the paper.

## Related literature

ICU admission and discharge control problems have been studied both by medical and management scholars. Several papers in medical journals (mostly based on retrospective empirical analyses) demonstrate that both delayed admission and demand-driven early discharge result in negative medical outcomes. Chalfin et al. [26] state that patients should be admitted to the ICU as soon as possible, as rejections or delays lead to undesirable consequences. There are plenty of studies discussing the effects of early discharge and readmission in the medical literature. The researchers agree that patients discharged early face additional risks of health deterioration, which might lead to readmission to the ICU. A few studies indicate that these patients tend to have higher mortality than first-time admitted patients [27–29]. To monitor the time to readmission, Helm et al. [30] estimate a readmission density function in order to optimize a post-discharge monitoring schedule and staffing plan. Furthermore, Chrusch et al. [31] conclude that high utilization levels of ICUs may increase readmission rates and mortality rates. Iapichino et al. [32] agree that higher occupancy levels (indicating higher severity levels) lead to higher mortality rates. Consistent with those studies, Bouneb et al. [33] find that bed availability is a main driver for ICU refusals, and that these refusals lead to an increase in mortality; Louriz et al. [34] report an increase of mortality levels of around 10pp (percentage points) in case of refused ICU admission.

Operations research/management science plays an important role in identifying ways to manage ICU capacity efficiently and in ensuring desired levels of service quality. An overview of the related literature concerning ICU management problems published since 1980 can be found in Bai et al. [7].

Several papers discuss the patient flow in ICUs by applying empirical approaches. KC and Terwiesch [15] analyze discharge and readmission processes with econometric statistical methods. They demonstrate that early discharging ICU patients leads to higher ICU readmission rates. Focusing on patients admitted via the ED, Kim et al. [35] evaluate the effect of ICU admissions on patient outcomes by analyzing a large dataset. They conclude that the admission probability is strongly impacted by ICU capacities – the probability of being admitted significantly decreases with increasing ICU utilization. They demonstrate that admitting patients has preferable outcomes; for instance, readmissions or transfers can be significantly decreased. Thus, admission policies might have a considerable impact on patient outcomes. Based on their empirical findings, they model the admission control problem as a discrete version of the Erlang loss model, similar to Shmueli et al. [36], and apply a simulation to estimate the benefit of alternative admission policies. A threshold rule that leads to admission of patients based on the health status and the remaining free capacities shows promising results – the benefits of applying such a policy clearly exceed those of creating an additional bed. Hu et al. [37] focus on ICU admission decisions of internal emergency patients using a data set of 21 hospitals. While they find that early admissions of internal emergencies can significantly reduce negative medical consequences such as mortality, admitting patients proactively can also congest ICUs, leading to an increase of early discharges. A study focusing on the effects of occupancy levels on ICU LOS is carried out by Long and Mathews [38]. They divide the time a patient occupies an ICU bed in a real “service time”, where care is provided, and a “boarding time”, where patients are basically ready to leave but wait to be discharged. This boarding time correlates with occupancy levels of both hospital wards and the ICU – it increases with increasing ward occupancy, and decreases with increasing ICU occupancy. Interestingly, the effect of high ward occupancy seems to overweight the effect of high ICU occupancy, as in those situations, long boarding times are observed. Miedaner and Sülz [39] study 18 German neonatal intensive care units to analyze whether the ICUs should have a narrow focus and admit a homogeneous patient cluster or whether they should admit a pool of patient clusters. With an empirical study, they found that the organizational units providing services for complex patients should not have a narrow focus, but should rather provide services for related patient segments.

In the analytical domain, queueing theory and Markov models are the methods mostly applied to ICU admission and discharge control problems. Three of these models apply different variations of queuing theory: Griffiths et al. [40] model the ICU admission control problem as an M/H/c/∞/FIFO (first-in-first out) model, and similarly, Kim et al. [41] apply an M/M/c multi-server system to analyze admission control processes. Shmueli et al. [36] apply a similar M/M/c model to compare myopic first-come-first-served policies to those where only patients with a certain incremental benefit are admitted. They demonstrate that higher rejection rates can lead to preferable medical outcomes. Finally, Chan and Yom-Tov [42] set up an Erlang-R queueing model to make discharge decisions.

MDP plays an important role not only for ICUs, but also for various other hospital departments, such as operating rooms, EDs, and inpatient wards. Barz and Rajaram [43] use an MDP for admission control in a hospital. To accept emergency patients under multiple resource constraints, they decide whether to accept or reject elective patients. Approximate dynamic programming-based heuristics are used to solve the model. Samiedaluie et al. [44] study the admission policies in a neurology ward by an infinite horizon dynamic programming approach. Multiple types of patients are classified based on their medical characteristics. The large scale case study solved by approximate dynamic programming (ADP) prove that the optimal policies can reduce the overall deterioration in patients’ health status. Zonderland et al. [45] develop an MDP-based decision support tool to schedule the admission of elective and semi-urgent surgeries, considering the capacity of operating rooms. Similarly, Yang et al. [46] optimize the admission policy for surgery patients considering capacity constraints in the surgical ICU. The patients are grouped based on the surgeon performing the surgery. They apply a heuristic solution method to solve the MDP. Even in regular wards, hospitals face the problem of insufficient capacity. Thompson et al. [4] manage ward capacity by transferring patients between different floors in the hospital. To optimize floor choice, they develop and implement an MDP-based decision support system. Gocgun and Puterman [47], Gupta and Lei [48] and Yu et al. [49] apply an MDP appointment scheduling model to optimize the utilization of medical resources, and also solve it using approximate dynamic programming. Li et al. [50] apply dynamic programming as well to schedule limited resources to a large number of jobs. Xie et al. [51] implement a nested policy based on dynamic programming solutions to schedule the appointments for a medical diagnostic facilities.

Four papers using Markov models in an ICU context are most closely related to our work. Dobson et al. [52] use a Markov chain model to evaluate ICU performance of an exogenously given, intuitive decision rule. They model time as discrete days and define patients by their remaining LOS, which, they argue, is in reality deterministic for most patients. If a patient arrives at a full ICU, the one with the shortest remaining LOS (even possibly the new arrival) is discharged early. Chan et al. [12], Li et al. [53], and Li et al. [14] use finite horizon, discrete time MDPs to derive optimal ICU policies. More specifically, Chan et al. [12] consider a planning horizon of one week and use a state space containing the number of patients of several types that are in the ICU. These patient types are defined by their expected initial LOS that is determined by a patient’s condition when he/she enters the ICU. Patients do not change their type and the types have different probabilities for a regular discharge in one time period. In each time period, the decision problem is whether and which patient to discharge early. They only briefly discuss rejections of external emergency patients, but suggest in their outlook the consideration of ICU admission decisions to enable a more holistic view. In contrast, Li et al. [53] study ICU admission decisions with a planning horizon of one day. Because of this short horizon, they assume that patients’ health conditions do not change and there are no regular discharges. They distinguish two patient types based on the initial health status. The health condition of type 1 patients is more severe (diagnosed with sepsis, respiratory failure, or problems with the central nervous system) and they are always admitted, even if a (healthier) type 2 patient must be discharged early because the ICU is full. Type 2 patients may be admitted if there are free beds. However, if a type 2 patient is first admitted and later early discharged, it would have been better not to admit him/her. Thus, the decision problem considered is whether to accept an arriving type 2 patient given the current state of the ICU. The authors show that a threshold-type policy is optimal, that is, type 2 patients are only admitted if a certain number of beds is free and that this threshold decreases over time. However, this decrease is obviously an artefact of the artificially limited planning horizon. Controlling for start- and end-of-horizon effects, a time-homogeneous problem probably features a stationary solution. Threshold-based policies are often observed in real-life ICU decision making. Li et al. [14] focus on the maximization of the survival benefits by optimizing the ICU planning with early discharge from an engineering perspective. Their classification of patients follows Li et al. [53]. Although a longer time horizon of ten days is considered, patients still do not change their health status. Unfortunately, there are some disconnects between text and model (e.g. the probability of any health status change is independent of the ICU occupation), which may be caused by the need for simplifications to enable the analytical derivation of structural properties. Surprisingly, the optimal policy derived implies some situations where only the less critical type 2 patients are admitted, but the more critical type 1 patients are rejected.

The papers that are most connected to our work are Kim et al. [35] in the empirical literature, and Li et al. [53] and Li et al. [14] in the modeling literature. We see our approach as complementary to Kim et al. [35]. While they analyze a huge dataset to derive information on admission policies and consequences of those, our approach analytically models such policies. Contrary to Kim et al. [35], our model does not focus on patients admitted via the ED only, but also includes patients with scheduled surgeries or internal emergencies. Compared to the last two papers mentioned above, our model is based on less restrictive assumptions to capture important problem characteristics. In particular, our state space contains patients’ current health status (high- or low-severity), and, thus, we consider health status changes while staying in the ICU: some patients recover and some get worse. Furthermore, in line with Chan et al. [12], we derive the probability of regular discharges from the current patient mix in the ICU. Finally, none of the papers discussed above considers the effects of medical and monetary goals, and the underlying trade-off decisions.

## Problem description and model formulation

Both admission control and demand-driven early discharge decisions are included in the MDP model. In case a patient arrives at a congested ICU, there are two possible options: to reject the new patient and to discharge an existing patient early to make room for the new patient. However, both options can lead to negative consequences. Therefore, our objective is to find the optimal decision policy in order to minimize negative consequences of capacity shortage, which can be assessed from a medical or monetary perspective.

### Problem setting

**Fig. 2:**
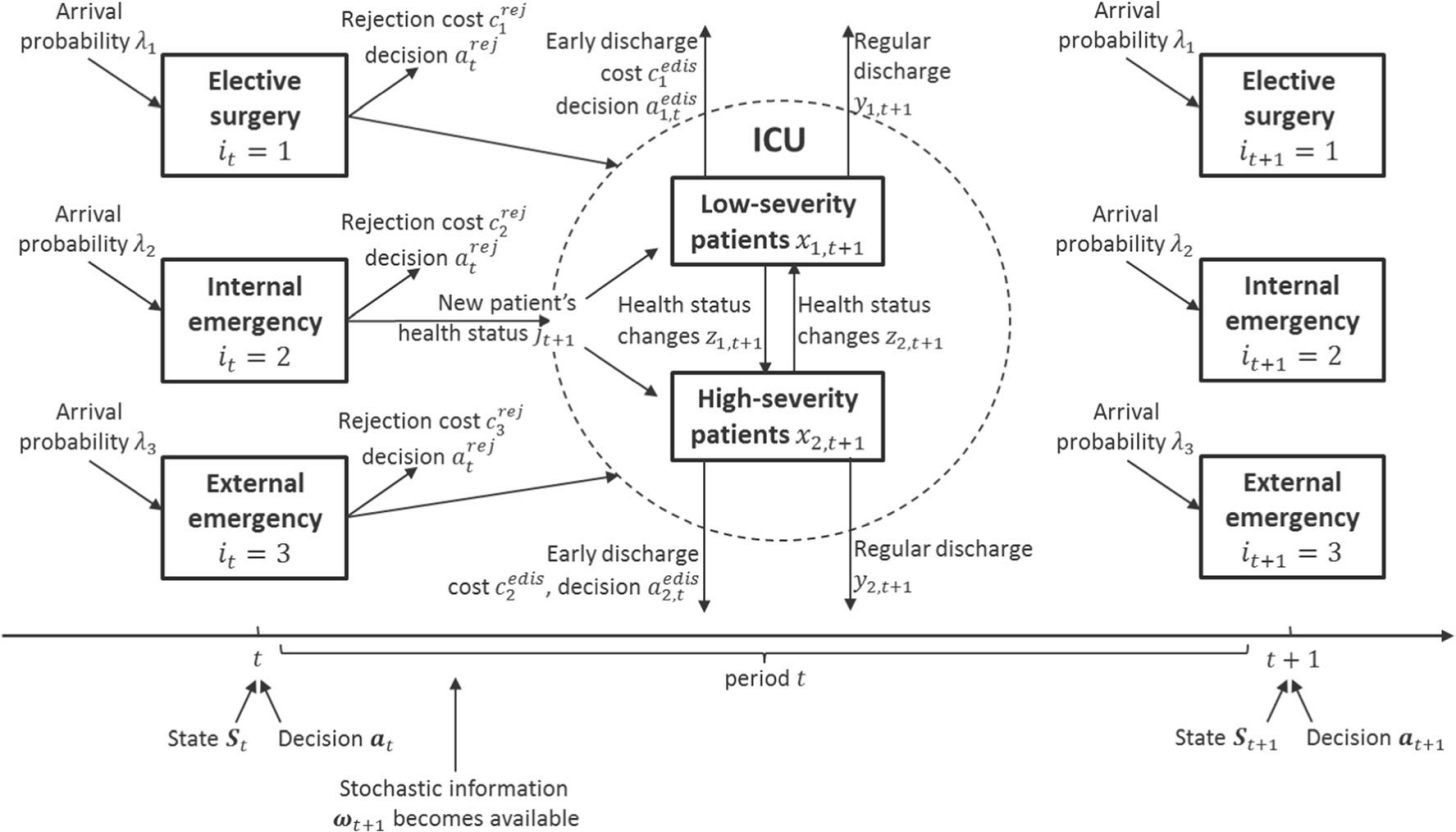
Sequence of events

We model the problem as a (stationary) discrete time Markov decision process as illustrated in Fig. 2. The objective is finding the admission and discharge policy that minimizes average total cost.

We assume an infinite time horizon, and define a time period small enough that at most one patient arrives within each time period. The sequence of events is as follows: At time *t* ∈ {1, 2…}, time period *t* begins and all information indexed with *t* is available. The ICU with a total capacity of *B* beds is occupied by *x*_*j*,*t*_ low-severity (*j* = 1) and high-severity (*j* = 2) patients and a new patient of type *i* ∈ {1, 2, 3} (elective surgery, internal emergency, and external emergency) just arrived. If no patient arrived, we set *i* = 0. Now, admission of this patient and early discharge of an existing patient are decided. Please note that deciding on an arriving patient is for illustration purposes only. These decisions are equivalent to the hospital deciding in advance what it would do with an arriving patient. In practice, electives as well as external emergencies would not arrive and be rejected but would rather be canceled or diverted in advance.

The decisions are captured by the binary action vector ${\boldsymbol{a}}_t=\left({a}_t^{rej},{a}_{1,t}^{edis},{a}_{2,t}^{edis}\right)$ whose elements indicate rejecting the arriving patient and early discharging of a low-severity patient or a high-severity patient, respectively. Rejecting a type *i* arrival leads to penalty costs of ${c}_i^{rej}$ and early discharging an existing patient of health status *j* costs ${c}_j^{edis}$. Since the bed preparation time for new patients is relatively short, we ignore it for new patients as current ICU modeling literature suggests [12, 14, 54]. Therefore, an early discharge can make room for a new patient. As mentioned before, only after a new patient is admitted to the ICU, his/her health status *j*_*t* + 1_ ∈ {1, 2} becomes known. This is because if the patient was not admitted, for example, the ambulance would be diverted and we would never know about that. Furthermore, both types of patients can be regularly discharged.

Moreover, patients’ health status may change. A number *y*_*j*,*t* + 1_ of patients are regularly discharged and *z*_*j*,*t* + 1_ patients change their status from *j* to (3 − *j)*. Technically speaking, the new information ***ω***_*t* + 1_ = (*j*_*t* + 1_,  *y*_1,*t* + 1_,  *y*_2,*t* + 1_, *z*_1,*t* + 1_, *z*_2,*t* + 1_, *i*_*t* + 1_) becomes available. If the current patient is admitted, his/her health status *j*_*t* + 1_ is observed. The information also includes the possible arrival of the next patient *i*_*t* + 1_. In the following, we present the elements of the MDP model in detail. All parameters and variables of the model are listed in Table 1.

**Table 1:** Parameters and variables of the MDP model

Patient indices	*i*: arriving patient’s type (*i* = 1: elective surgery; *i*=2, internal emergency; *i* = 3, external emergency; *i* = 0, no arrival) *j*: index for health status: (*j* = 1, low-severity; *j* = 2, high-severity)
Cost parameters	${c}_i^{rej}$: rejection cost of a patient type *i* ∈ {1, 2, 3} (when the request arrives, only the arrival type is known) ${c}_j^{edis}$: early discharge cost of patient with health status *j* ∈ {1, 2} $\boldsymbol{c}=\left({c}_1^{rej},{c}_2^{rej},{c}_3^{rej},{c}_1^{edis},{c}_2^{edis}\right)$: cost vector
Distribution parameters	**h** = [*h*_*i*, *j*_]_2 × 3_: probabilities that a type *i* patient has health status *j* if admitted ${p}_j^{dis}$: probability that a given patient of status *j* is regularly discharged in one period ${p}_j^{cha}$: probability that a given patient of status *j* changes the health status in one period λ_*i*_: probability that a type *i* patient arrives, no arrival with probability *λ*_0_ = 1 − ∑_*i*_*λ*_*i*_
Other parameters	*B*: total capacity of ICU (number of beds) *T*: length of time horizon (index *t* ∈ {1, …, *T*})
Action variables	${a}_t^{rej}$: binary decision variable indicating whether to reject the arriving patient (${a}_t^{rej}=1$) ${a}_{j,t}^{edis}$: binary decision variable indicating whether to early discharge a patient with health status *j* (${a}_{j,t}^{edis}=1$) ${a}_t=\left({a}_t^{rej},{a}_{1,t}^{edis},{a}_{2,t}^{edis}\right)={A}^{\pi}\left({\boldsymbol{S}}_t\right)$: action vector decided at time *t* with policy *π*
State variables	*x*_*j*,*t*_: number of patients with health status *j* in ICU at time *t* *i*_*t*_: the arrival type of the new patient at period 푡 ***S***_*t*_ = (*x*_1, *t*_, *x*_2, *t*_, *i*_*t*_): state vector at period *t*
Stochastic information	*j*_*t* +1_: health status of new patient (a patient of type *i* has health status *j* with probability *h*_*i*, *j*_, known if admitted in *t*) *y*_*j*,*t*+1_: number of regular discharges of type *j* patients during period *t*. ${y}_{j,t+1}\sim B\left({x}_{j,t}-{a}_{j,t}^{edis},{p}_j^{dis}\right)$ *z*_*j*,*t* + 1_: number of patients of type *j* patients who change their health status during period *t*. ${z}_{j,t+1}\sim B\left({x}_{j,t}-{a}_{j,t}^{edis},{p}_j^{cha}\right)$ *i*_*t* +1_: the arrival type of the new patient at period *t* + 1 (a patient is of type *i* with probability *λ*_*i*_) ***ω***_*t* + 1_ = (*j*_*t* + 1_, *y*_*j*, *t* + 1_, *z*_*j*, *t* + 1_, *i*_*t* + 1_): vector of information that becomes available at the end of period *t*

### State, action and policy

We use the pre-decision state which captures the state of the system immediately before a decision is taken. The *state* ***S***_*t*_ = (*x*_1,*t*_, *x*_2,*t*_, *i*_*t*_) at the beginning of time period *t* is defined by three elements: the number of low-severity (*x*_1,*t*_) and high-severity (*x*_2,*t*_) patients in the ICU as well as the type of the arriving patient *i*_*t*_. We set *i*_*t*_ = 0 if there is no arrival and assume that while the arrival type is known at arrival, the health status can only be diagnosed when the patient is admitted at the ICU.

The *action vector* ${\boldsymbol{a}}_t=\left({a}_t^{rej},{a}_{1,t}^{edis},{a}_{2,t}^{edis}\right)$ consists of binary elements indicating whether the arriving patient is rejected (${a}_t^{rej}=1$), as well as whether a low-severity patient (${a}_{1,t}^{edis}=1$) or a high-severity patient (${a}_{2,t}^{edis}=1$) is discharged early. We are interested in a decision rule or *policy π* that gives a best action ***a***_*t*_ for every state ***S***_*t*_. Thus, the action is a function of the state: ***a***_*t*_ = *A*^*π*^(***S***_*t*_).

### Stochastic events, transformation function and transition probabilities

*Stochastic events* include four parts. During period *t*, the information ***ω***_*t* + 1_ = (*j*_*t* + 1_, *y*_1, *t* + 1_, *y*_2, *t* + 1_, *z*_1, *t* + 1_, *z*_2, *t* + 1_,  *i*_*t* + 1_) becomes available. If patient *i*_*t*_ was admitted to the ICU, his/her health status *j*_*t* + 1_ ∈ {1, 2} becomes known. A number *y*_*j*, *t* + 1_ of patients is regularly discharged and *z*_*j*, *t* + 1_ patients change their health status, for *j* = 1, 2. Finally, a new patient *i*_*t* + 1_ might arrive.

The new state ***S***_*t* + 1_ at the beginning of the next period *t* + 1 is a function of the previous state ***S***_*t*_, the action ***a***_*t*_ and the new information ***ω***_*t* + 1_. It is given by the following *transformation function*, which could be easily generalized to more health statuses (please note that sgn(*i*_*t*_) takes a value of 1 if a patient arrives (*i*_*t*_ > 0), and 0 if no patient arrives (*i*_*t*_ = 0)):

1 $$ {\boldsymbol{S}}_{t+1}\left({S}_t,{\boldsymbol{a}}_t,{\boldsymbol{\omega}}_{t+1}\right)=\left(\begin{array}{c}{x}_{1,t}+\operatorname{sgn}\left({i}_t\right)\dot{\mkern6mu}\left(1-{a}_t^{rej}\right)\dot{\mkern6mu}\left(2-{j}_{t+1}\right)-{a}_{1,t}^{edis}-{y}_{1,t+1}-{z}_{1,t+1}+{z}_{2,t+1},\\ {}{x}_{2,t}+\operatorname{sgn}\left({i}_t\right)\dot{\mkern6mu}+\left(1-{a}_t^{rej}\right)\dot{\mkern6mu}\left({j}_{t+1}-1\right)-{a}_{2,t}^{edis}-{y}_{2,t+1}-{z}_{2,t+1}+{z}_{1,t+1},\\ {}{i}_{t+1}\end{array}\right). $$

Both the patients’ arrivals and LOS contain uncertainties that are difficult to model. Although some researchers argue that there are no suitable distributions to model the arrival pattern and LOS in the ICU [55], and especially the LOS is not reliably predictable for individual patients [8], most papers in the literature apply theoretical distributions. We apply memoryless distributions for arrival rates and lengths of stay, an assumption that proved to be suitable in the literature [7].

Regarding the elements of ***ω***_*t* + 1_, we model the following dependencies and distributions:

- The new patient’s health status *j*_*t* + 1_ depends on his/her type *i*_*t*_. Parameters *h*_*i*, *j*_ give the probability that a type *i* patient has health status *j* if admitted and are grouped into a matrix **h** = [*h*_*i*, *j*_]_3 × 2_. In case a patient is not admitted, the status is meaningless and, technically, an arbitrary one realizes.
- The number of regular discharges *y*_*j*, *t* + 1_ depends on the number of type *j* patients in the ICU, that is, ${x}_{j,t}-{a}_{j,t}^{edis}$. Each patient is regularly discharged (including transfers and events of death) with ${p}_j^{dis}$, independently from the other patients. Thus, the number of regular discharges *y*_*j*, *t* + 1_ follows a binomial distribution: ${y}_{j,t+1}\sim B\left({x}_{j,t}-{a}_{j,t}^{edis},{p}_j^{dis}\right)$.
- Analogously, the number of patients who change their status *z*_*j*, *t* + 1_ depends on the number of patients in the ICU as well, that is, also ${x}_{j,t}-{a}_{j,t}^{edis}$. Each patient’s health status improves or deteriorates with probability ${p}_j^{cha}$ in one period, independent of the other patients. Thus, *z*_*j*, *t* + 1_ follows a binomial distribution: ${z}_{j,t+1}\sim B\left({x}_{j,t}-{a}_{j,t}^{edis},{p}_j^{cha}\right)$. In addition, the sum of regular discharges *y*_*j*, *t* + 1_ and patients who change their status *z*_*j*, *t* + 1_ cannot exceed the number of patients in the ICU, that is, ${x}_{j,t}-{a}_{j,t}^{edis}\ge {y}_{j,t+1}+{z}_{j,t+1}$
- Finally, with probability *λ*_*i*_, a new patient of type *i* arrives and with probability *λ*_0_ = 1 − ∑_*i*_*λ*_*i*_, there is no arrival. We group these into the parameter vector ***λ*** = (*λ*_1_, *λ*_2_, *λ*_3_).

Thus, the stochastic distributions are described by the following set of parameters: $\mathbf{h},{p}_j^{dis},{p}_j^{cha},\boldsymbol{\lambda}$.

### Cost function and value function

The one-step *cost function C*(***S***_*t*_, ***a***_*t*_) captures the cost of decision ***a***_*t*_ in state ***S***_*t*_:

2 $$ C\left({\boldsymbol{S}}_{\boldsymbol{t}},{\boldsymbol{a}}_t\right)=\left\{\begin{array}{c}{c}_{i_t}^{rej}\dot{\mkern6mu}{a}_t^{rej}\dot{\mkern6mu}\operatorname{sgn}\left({i}_t\right)+{\sum}_j{c}_j^{edis}\dot{\mkern6mu}{a}_{j,t}^{edis}\kern0.5em \\ {}\infty \kern0.5em \end{array}\begin{array}{l}, if\ {x}_{1,t},{x}_{2,t}\ge 0\wedge {x}_{1,t}+{x}_{2,t}\le B\\ {}, otherwise\end{array}\right.. $$

Rejecting a type *i* arrival leads to penalty costs of ${c}_i^{rej}$ and discharging an existing patient of health status *j* early costs ${c}_j^{edis}$. Note that the first line of (2) refers to feasible states. The second line prevents that an action is chosen that leads to an infeasible state via costs of infinity (for example, more than *B* beds occupied). We group the cost parameters into the vector $\boldsymbol{c}=\left({c}_1^{rej},{c}_2^{rej},{c}_3^{rej},{c}_1^{edis},{c}_2^{edis}\right)$. The “costs” in this model are an abstract concept, and its implications depend on the “cost” perspective applied. For instance, costs could be defined to be the negative effects to the patient health condition, or lost profits from a monetary perspective. Now, we can define the objective function. As we minimize average total cost, this is

3 $$ {V}_t\left({\boldsymbol{S}}_t\right)=\underset{\pi }{\min}\underset{T\to \infty }{\lim}\frac{1}{T}{\mathbbm{E}}_{\omega}\left[\sum \limits_{t=1}^TC\left({\boldsymbol{S}}_t,{A}^{\pi}\left({S}_t\right)\right)\right] $$

with ***S***_*t*_ = ***S***_*t* + 1_(***S***_*t*_, *A*^*π*^(***S***_*t*_), ***ω***_*t* + 1_).

## Model input: Medical and monetary perspective of admission and discharge consequences

Based on historical data from a large German teaching hospital and the current literature, we estimate model parameters, namely a set of distribution parameters and the cost parameter vector ***c***. The used patient-related data is either anonymized data or aggregated data, not requiring any patient informed consent in accordance with the European General Data Protection Regulation (EU directive - 2016/679). In Subsection 4.1, we analyze patient arrivals and the evolution of their health status (corresponding to the lengths of their stays) and derive the distribution parameters. In Subsection 4.2, we consider the cost vector ***c*** based on two different objectives of optimization, namely a medical and a monetary perspective.

### Analysis of historical arrival and LOS data

We obtained three months’ worth of data concerning patient arrivals and discharges within an ICU of a large German teaching hospital. There are in total *B* = 35 beds in this ICU, and 514 patients were admitted during this time period. For each patient, we know his/her arrival type and LOS. Arrivals are highly fluctuating and range from 1 to 12 patients per day. The utilization level is high (about 95%).

We define the length of a time period as one hour, so that we can assume that there is at most one arrival per period. In the following, we shortly sketch how we obtained the required parameters $\mathbf{h},{p}_j^{dis},{p}_j^{cha},\boldsymbol{\lambda}$ from real-life data.

#### Arrival process

We analyze the historical data and conclude that Poisson processes are adequate to describe the arrival process for all three patient types. However, in the historical data, we know only the number of admitted patients without any records on the number of rejected patients. According to the literature [56–58], the percentage of patients being denied admission to the ICU ranges between 20% and 60%. Consistent with McManus et al. [59], who analyzed rejection rates in relation to ICU utilization, we increase the historical admission numbers by a factor of 1.25 (that is, assuming an admission rate of 0.8) to calculate the arrival probabilities. In each time period, there can be an arrival of an elective surgery, an internal emergency patient, an external emergency patient, or no arrival. As we have the arrival type in the data, we can directly calculate the following probabilities:

$$ \boldsymbol{\lambda} =\left({\lambda}_1,{\lambda}_2,{\lambda}_3\right)=\left(0.088,0.153,0.059\right)\ \mathrm{and},\mathrm{accordingly},{\lambda}_0=0.700. $$

**Fig. 3:**
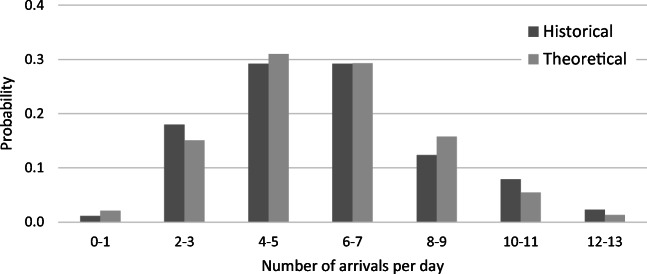
Comparison of historical and theoretical arrival process

A visual comparison of historic arrivals and the theoretical predictions (before increasing the parameters by 1.25) is illustrated in Fig. 3.

#### Health status evolution

**Fig. 4:**
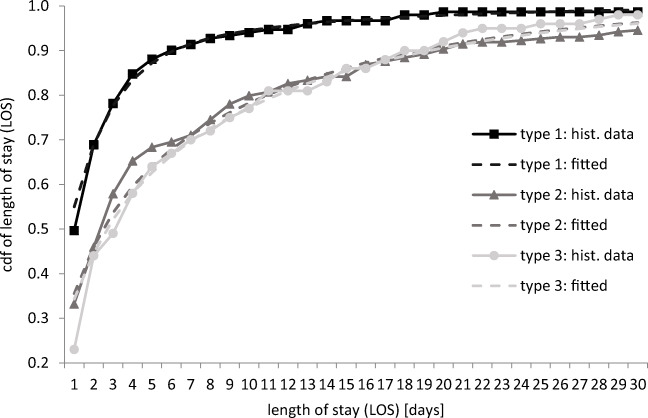
Comparison of historical and theoretical LOS distribution

In a first step, we directly determined the empirical distribution of the LOS for each patient type from the data (solid lines in Fig. 4). In a second step, we calibrated the stochastic model outlined in Section 3.3 to these distributions. Figure 4 shows a good fit between the historical (solid lines) and the theoretical (dashed lines) LOS distributions.

More technically, we used a grid search to choose the probabilities $\mathbf{h},{p}_j^{dis}$, and ${p}_j^{cha}$ for each patient type such that the resulting probability distribution function of the LOS distribution most closely resembles the empirically observed one. Distance was defined as the sum of the absolute distances for each day. In doing so, we again assume that all patients are admitted (as only this is contained in our data) and no early discharges occurred. We obtained the following distribution parameters:

$$ \mathbf{h}=\left[\begin{array}{cc}0.9980& 0.002\\ {}0.5426& 0.4574\\ {}0.5141& 0.4859\end{array}\right],{p}_j^{dis}=\left[\begin{array}{c}0.0177\\ {}0.0024\end{array}\right],{p}_j^{cha}=\left[\begin{array}{c}0.0019\\ {}0.0014\end{array}\right],\kern0.5em \boldsymbol{\lambda} =\left(0.088,0.153,0.059\right). $$

Thus, the share of high-severity patients depending on the arrival type is as follows: *h*_1, 2_ = 0.2% of elective surgery patients, *h*_2, 2_ = 45.74% of internal emergency patients, and *h*_3, 2_ = 48.59% of the external emergency patients are high-severity patients. Note that most elective surgeries result in low-severity patients.

The probability of a regular discharge in the next period is ${p}_1^{dis}=1.77\%$ for a low-severity patient and ${p}_2^{dis}=0.24\%$ for a high-severity patient. In our case study, the capacity of the ICU is *B* = 35 beds. No matter how many low-severity patients are in the ICU, the probability of regularly discharging more than three low-severity patients is below 0.3%. Therefore, to simplify the solution of the MDP in this case study, we only consider three or less regular discharges in each time period, that is *y*_1, *t* + 1_ ≤ 3. With the same logic, we find that the probability of regularly discharging more than one high-severity patient is below 0.3%. Therefore, we can assume that at most one high-severity patient is regularly discharged, i.e. *y*_2, *t* + 1_ ≤ 1. This considerably reduces the number of state transitions to consider without simplifying too much. Of course, these simplifications depend on ICU size and the probabilities. If the ICU capacity is orders of magnitude bigger, then considering 2 or more simultaneous discharges may be necessary. But based on our knowledge, we feel this assumption should be widely applicable.

The probability that a low-severity patient worsens to high-severity is ${p}_1^{cha}=0.19\%$, while the probability that a high-severity patient improves to low-severity is ${p}_2^{cha}=0.14\%$. Again, we analyze the probabilities for all possible numbers of health status changes. For example, it can be shown for our data set that the probability of *z*_2, *t* + 1_ health status changes from high- to low-severity is highest if the ICU is full of high-severity patients (${x}_{2,t}-{a}_{2,t}^{edis}=35$) and decreases in *z*_2, *t* + 1_ for our data. For *z*_2, *t* + 1_ = 2, it is only 0.2%. On the contrary, when ${x}_{1,t}-{a}_{1,t}^{edis}=35$, the probability of *z*_1, *t* + 1_ = 2 is 0.1%. Thus, to simplify the computation of the state transitions, we assume that at most 1 patient of each type (*z*_*j*, *t* + 1_ ≤ 1) will change the health status during one time period.

### Definition of costs

Our model minimizes the costs, that is, negative consequences of capacity shortages within the ICU. Obviously, there is no global definition of negative consequences. In the following, we define two possible perspectives: A medical perspective that minimizes the increase of mortality rates, and a monetary perspective that minimizes the negative effects on hospital profits due to lost revenues and additional costs. This offers the opportunities to discuss the value of our MDP approach compared to myopic heuristics in both perspectives, the consequences of optimizing the medical perspective on monetary performance indicators and vice versa, and possible structures of systems where both perspectives are aligned.

Contrary to lengths of stays and arrival rates as discussed in Section 4.1, data on the direct consequences of capacity shortages on mortality rates or hospital profits are typically not available. Besides, they depend on the specific case: mortality rates depend on the level of care and the patient mix. Effects on profits depend on the reimbursement system and contractual specifications. For our case study, we chose the following approach: Regarding the medical perspective, we derive realistic ranges from the literature, and discuss the value for our case study with the case hospital’s ICU manager. Regarding the monetary perspective, we rely on the Diagnosis Related Groups system of Germany for the year 2017, the same year which is relevant for our hospital case study. This system publishes cost components based on 1,144 diagnosis groups covering 2.5 million patients. Thus, possible revenues and costs for treatments covering surgeries or intensive care can be derived. In case of rejecting internal emergencies or discharging patients early, additional nursing care might be required. Here, we rely on full cost averages for nurses in Germany. As specified in the previous section, ICU management may choose five possible actions – each with an associated cost – to deal with capacity shortages, depending on the type of an arriving patient and the patients within the ICU. In the online appendix, we discuss how we derived the values for the cost vector $\boldsymbol{c}=\left({c}_1^{rej},{c}_2^{rej},{c}_3^{rej},{c}_1^{edis},{c}_2^{edis}\right)$ for the medical (***c***_*med*_) in Appendix B.1 and the monetary perspective (***c***_*mon*_) in Appendix B.2. The selected cost vectors for the medical and the monetary perspective are as follows:

$$ {\boldsymbol{c}}_{med}=\left({c}_{1, med}^{rej},{c}_{2, med}^{rej},{c}_{3, med}^{rej},{c}_{1, med}^{edis},{c}_{2, med}^{edis}\right)=\left(1\mathrm{pp},15\mathrm{pp},3\mathrm{pp},2\mathrm{pp},10\mathrm{pp}\right). $$

$$ {\boldsymbol{c}}_{mon}=\left({c}_{1, mon}^{rej},{c}_{2, mon}^{rej},{c}_{3, mon}^{rej},{c}_{1, mon}^{edis},{c}_{2, mon}^{edis}\right)=\left(9,200\text{\EUR}, 5,800\text{\EUR}, 4,100\text{\EUR}, 700\text{\EUR}, 6,500\text{\EUR} \right). $$

Please note that a) the choice of parameter values might differ from hospital to hospital, and that b) some parameters might not be determined accurately. In the online appendix, we provide a detailed sensitivity analysis based on different parameter combinations (Appendix E.1) and parameter misspecification (Appendix E.2).

## Case study: Implications of admission and discharge policies

We tested the performance of our MDP-based approach and a myopic benchmark mimicking intuitive decision making in practice for both the medical and monetary perspective. In particular, we implemented the following two approaches:

1. *MDP*^*o*^ (*o* ∈ {med, mon}) is the optimal policy following from the MDP approach described in Section 3 optimizing the medical or monetary perspective, that is with the medical (***c***_*med*_) or monetary (***c***_*mon*_) cost vector. Technically, we used a finite horizon approximation with horizon *T* to calculate the stationary policy from the value function ${V}_t\left({\boldsymbol{S}}_t\right)=\underset{{\boldsymbol{a}}_t}{\min}\left\{\frac{1}{T-t+1}\dot{\mkern6mu}C\left({\boldsymbol{S}}_t,{\boldsymbol{a}}_t\right)+\frac{T-t}{T-t+1}\dot{\mkern6mu}{\mathbbm{E}}_{{\boldsymbol{\upomega}}_{\mathrm{t}}}\left[{V}_{t+1}\left({\boldsymbol{S}}_{t+1}\left({S}_t,{\boldsymbol{a}}_t,{\boldsymbol{\omega}}_{t+1}\right)\right)\right]\right\}$ with the boundary condition *V*_*T* + 1_(***S***_*T* + 1_) = 0. This MDP was first solved via backwards induction (see Appendix A), which involves the calculation of $\frac{\left(B+1\right)\left(B+2\right)}{2}.4$ states per time period, that is 2664 states for *B* = 35. The time horizon was sufficiently large such that the ICU is in a steady state in the first time periods and, therefore, the optimal policy reported here does not depend on the time period. In our experiments, we used *T* = 168 and observed time-independent actions for the first 5 time periods. The runtime for this one-week horizon was about 30 min without parallelization, which is negligible, as the optimization needs to be run only once for a set of parameters.
2. *Myopic*^*o*^ (*o* ∈ {med, mon}) is the benchmark policy following from an intuitive, hands-on approach. This policy mimics the decision making in practice and reflects the status quo in our case study hospital. It only takes immediate costs into account. For example, Chan et al. [11] applied a similar myopic heuristic. More precisely, as opposed to the minimization of immediate *C*(***S***_*t*_, ***a***_*t*_) and future costs ${\mathbbm{E}}_{\omega_{\mathrm{t}}}\left[{V}_{t+1}\left({\boldsymbol{S}}_{t+1}\left({S}_t,{\boldsymbol{a}}_t,{\boldsymbol{\omega}}_{t+1}\right)\right)\right]$ in the MDP’s value function (3), *Myopic* minimizes only *C*(***S***_*t*_, ***a***_*t*_). In our setting, this results in the following simple decision rules:
  *Decision rule 1*: If no patient arrives, do nothing.
  *Decision rule 2:* If there are free beds, accept any arriving patient without early discharging.
  *Decision rule 3:* If there is no free bed, select the cheapest alternative among rejecting the arriving patient or early discharge of a low-/high-severity patient from the ICU.

All policies were evaluated by simulation for a one-year horizon comprising 8760 1-h time periods with randomly generated arrivals, health status changes, etc. To eliminate start-of-horizon effects, we simulated an additional 1000 time periods before this evaluation horizon because preliminary tests showed that after about 600 time periods, start-of-horizon effects were not visible any more. The values reported are averages over 1000 simulation runs. Wherever appropriate, we also state the 95% confidence intervals of these means. The experiments were implemented using JAVA version 8 and ran on a computer with 3.20GHz CPU, 12 GB RAM, and 64-bit Windows 7.

### Policies resulting from the medical perspective

**Fig. 5:**
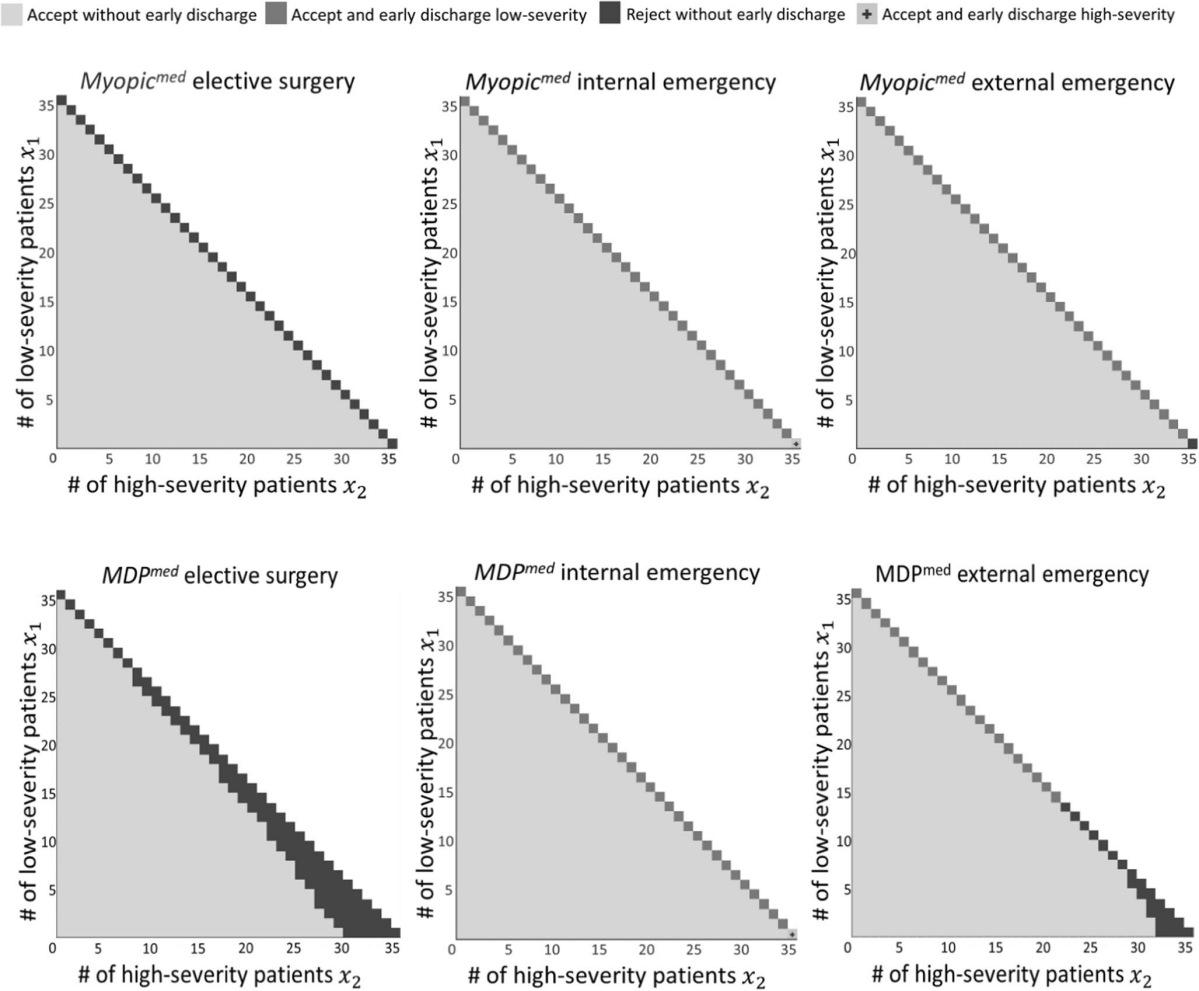
Comparison of the policies of *Myopic*^*med*^ (upper row) and *MDP*^*med*^ (lower row)

Figure 5 shows an overview of the policies resulting from *Myopic*^*med*^ (upper row) and *MDP*^*med*^ (lower row). For each possible state ***S*** = (*x*_1_, *x*_2_, *i*), it shows the action $\boldsymbol{a}=\left({a}^{rej},{a}_1^{edis},{a}_2^{edis}\right)$ taken. The columns represent the type of the arriving patient (*i* ∈ {1, 2, 3} for elective surgery, internal emergency, and external emergency, respectively). If no patient arrives, no action is taken, as discharging one of the existing patients early only has negative consequences and can be done later, if necessary. The axes represent the occupancy of the ICU. The vertical axis is the number of low-severity patients and the horizontal axis is the number of high-severity patients in the ICU. For example, the lower right square represents an ICU full of high-severity patients (*x*_1_ = 0, *x*_2_ = 35). Now, four policies representing the relevant combinations of actions exist (all other actions are dominated):

1. The patient is admitted, and no patient is early discharged (light shade).
2. The patient is admitted, and a low-severity patient is early discharged (medium shade).
3. The patient is admitted, and a high-severity patient is early discharged (bright shade with “+”).
4. The patient is rejected, and no patient is early discharged (dark shade).

Remember that the relationship of the rejection and early discharge costs in this scenario is ${c}_{i=1}^{rej}<{c}_{j=1}^{edis}<{c}_{i=3}^{rej}<{c}_{j=2}^{edis}<{c}_{i=2}^{rej}$. The *Myopic*^*med*^ policy is quite similar for all arrival types. In all cases, patients are admitted as long as empty beds exist (light shade below the diagonal). In case of a fully occupied ICU (represented by the diagonal), the action depends on the arriving patient: Elective surgeries (*i* = 1) will be always canceled, internal emergencies (*i* = 2) will always be admitted leading to early discharges (if possible, of low-severity patients), while external emergencies (*i* = 3) will be admitted if low-severity patients can be early discharged. In case only high-severity patients are in the ICU the external emergency patient will be rejected.

The *MDP*^*med*^ policy is identical for internal emergencies (*i* = 2), which are again always admitted. However, it differs for scheduled elective surgery patients (*i* = 1) and external emergencies (*i* = 3). In case the ICU contains many high-severity patients, these patients are rejected even if free beds exist. The effect is more pronounced for elective surgeries, from whom a free bed is already reserved even if only eight high-severity patients are in the ICU (*x*_1_ = 26, *x*_2_ = 8). In the most extreme case, with only high-severity patients in the ICU, elective surgeries will be canceled if no more than nine free beds exist, and external emergencies will be rejected if no more than four free beds exist. This is driven by the high cost of early discharging a high-severity patient and her/his longer expected stay in the ICU. Thus, even with some free beds, the MDP policy does not ‘risk’ having a full ICU in the future and rather rejects an elective surgery.

### Policies resulting from the monetary perspective

Second, we compare the myopic and MDP policies based on the monetary cost perspective (*Myopic*^*mon*^ and *MDP*^*mon*^). The relationship of the rejection and early discharge costs in this scenario is ${c}_{j=1}^{edis}<{c}_{i=3}^{rej}<{c}_{i=2}^{rej}<{c}_{j=2}^{edis}<{c}_{i=1}^{rej}$. Note that the rejection cost of an elective surgery patient is now the most expensive cost, while it was lowest in the medical perspective.

**Fig. 6:**
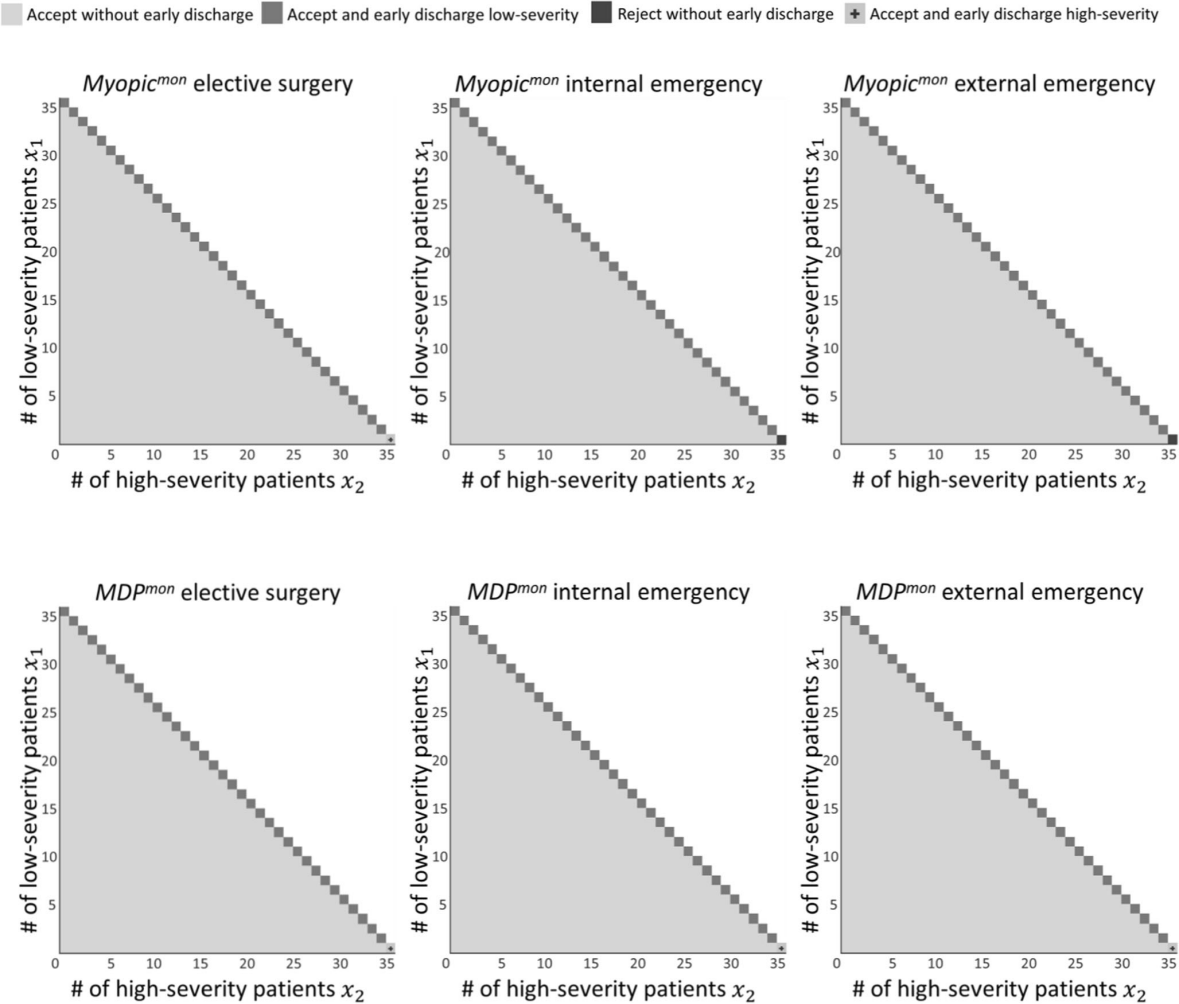
Comparison of the policies of *Myopic*^*mon*^ (upper row) and *MDP*^*mon*^ (lower row)

Both *Myopic*^*mon*^ and *MDP*^*mon*^ yield almost identical policies in this scenario (Fig. 6). If possible, all patients are admitted. If the ICU is full, the least critically ill patient is discharged early. The only exception is the case where only high-severity patients are in a full ICU, and an internal emergency (*i* = 2) or an external emergency patient (*i* = 3) arrives. While *Myopic*^*mon*^ rejects this patient, *MDP*^*mon*^ admits the patient and discharges a high-severity patient early.

### Performance analysis of admission and discharge policies

**Fig. 7:**
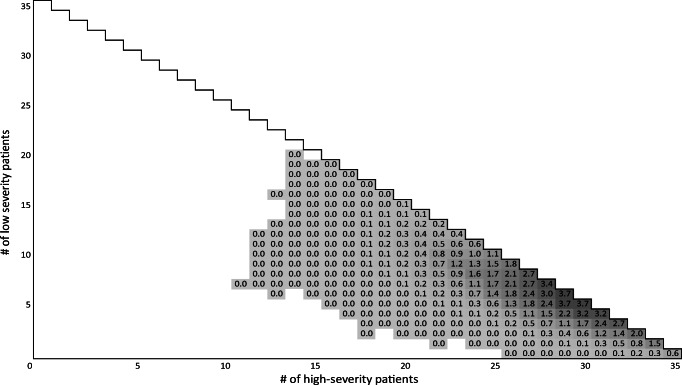
Frequency of ICU occupancy for *MDP*^*med*^ ([%]; empty/white: never observed after warm-up)

In this subsection, we analyze the performance of the policies described above over a one-year horizon using a simulation with 1000 runs. On average, 2628 patients arrived at the ICU during this year. We first describe ICU occupancy for *MDP*^*med*^ and *MDP*^*mon*^ using heatmaps and then share performance indicators like utilization and compare them to *Myopic*. The heatmap in Fig. 7 shows the relative frequency of the ICU occupancy using *MDP*^*med*^. Again, the vertical axis denotes the low-severity patients and the horizontal axis denotes the high-severity patients. Obviously, the ICU is never close to empty (the white area) and in the majority of time, around 1 to 10 low-severity and 23 to 34 high-severity patients are in the ICU (The 0.0 in the figure illustrates a probability below 0.1%). Overall, 82% of the patients in the ICU have the high-severity status, although they only account for roughly 20% of all admitted patients. This reflects the fact that high-severity patients stay longer. The key take-away here with regard to the interpretation of the policies shown in Fig. 5 is that an ICU full of high-severity patients (almost) never happens, but a full ICU with high-severity and low-severity patients is quite common (27.7%). There is frequently (41.3%) a high utilization level with only 1 or 2 free beds. Thus, the bed-reserving property by rejecting elective surgery patients that distinguishes *MDP*^*med*^ from *Myopic*^*med*^ is clearly relevant. However, bed-reserving by rejecting external emergencies does not create a large effect.

**Fig. 8:**
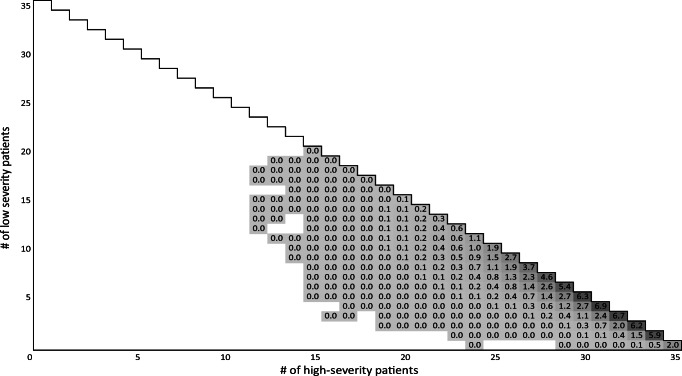
Frequency of ICU occupancy for *MDP*^*mon*^ ([%]; empty/white: never observed after warm-up)

The heatmap in Fig. 8 shows that a situation where *MDP*^*mon*^ admits the patient and early discharges a high-severity patient actually occurs in some events (in about 2.0% of the time periods, lower right square). Using *MDP*^*mon*^, the ICU is fully occupied in 54.6% of the time periods, more than twice as often as in the medical perspective. The ICU is almost full (1 or 2 free beds) in 33.4% of the time periods, thus, having more than 2 free beds is quite rare. We skip the visualization of the heatmaps for the myopic policies. As can be seen in Table 2, the resulting utilization levels are quite similar to *MDP*^*mon*^. Table 2 summarizes performance indicators for both approaches and perspectives together with their 95% confidence intervals. First, we discuss the monetary perspective, where myopic policies perform well, before we turn to the medical perspective, where the MDP policies lead to significant improvements.

**Table 2:** Comparison of performance indicators of *Myopic* and *MDP* (pp: percentage points)

Optimized Goal	Approach	Medical cost [pp]	Monetary cost [€]	Utilization rate [%]	Rejection rate [%]	Early discharge rate [%]
Medical	*MDP*^*med*^	1931 ± 197	7,160,950 ± 433,651	94.7 ± 0.6	32.6 ± 1.9	17.8 ± 2.5
	*Myopic*^*med*^	2453 ± 280	4,259,490 ± 420,612	97.4 ± 0.5	14.5 ± 1.3	38.6 ± 3.6
Monetary	*MDP*^*mon*^	2855 ± 319	1,143,772 ± 156,391	97.4 ± 0.5	0 ± 0	47.0 ± 3.6
	*Myopic*^*mon*^	3172 ± 412	1,239,946 ± 186,341	98.4 ± 0.4	2.0 ± 0.7	46.5 ± 3.3

In the monetary cost setting, the early discharge of low-severity patients has much lower costs compared to rejecting patients. Thus, both *MDP*^*mon*^ and *Myopic*^*mon*^ admit all patients and early discharge low-severity patients if necessary. Even though both policies are nearly identical (see Section 5.2), *MDP*^*mon*^ outperforms *Myopic*^*mon*^ regarding monetary cost by 7.8%.

However, this changes for the medical perspective. Here, *MDP*^*med*^ reserves more capacity for critical patients, and starts rejecting scheduled surgery and external emergency patients if too many high-severity patients are treated in the ICU (see Section 5.1). Thus, the average utilization is considerably lower compared to *Myopic*^*med*^ (94.7% versus 97.4%). Moreover, the effects differ: While the average increase in mortality due to capacity shortages is 2453pp per year for *Myopic*^*med*^, using *MDP*^*med*^ decreases this figure to 1931pp This means a reduction in additional annual mortality by 21% – thus, on average, 5.2 patients will die less every year due to the MDP policy.

Comparing the two objectives for the MDP policies (*MDP*^*mon*^ and *MDP*^*med*^), the differences are striking. Applying the monetary perspective, the one-year mortality due to capacity shortages rises from 1931pp to 2855pp, but the lost profits decrease from 7.1 million € to 1.1 million €. That is, the difference of those two objectives is losing around 9.2 patients’ lives against losing 6 million €. The reason for the mismatch between the medical and the monetary perspective lies within the reimbursement system.

**Fig. 9:**
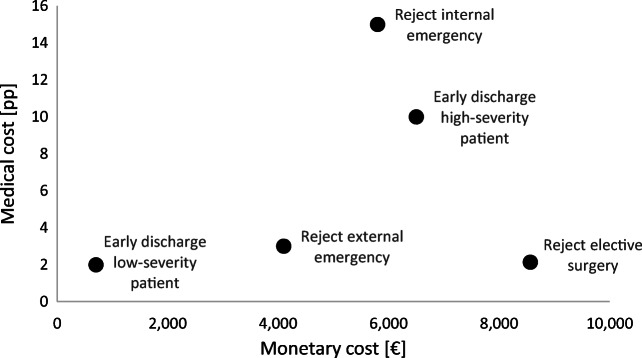
Medical and monetary costs for all five events

In Fig. 9, we plot the five events’ costs with the dimensions medical costs (additional mortality rate in pp) on the vertical axis and monetary costs (lost profits in Euro) on the horizontal axis. Medical and monetary consequences are aligned if they have a linear relationship. In our case, there is a major mismatch with regard to rejecting elective surgery patients. This action is at the same time the most favorable from a medical perspective and the least favorable from a monetary perspective. A smaller mismatch occurs with the rejection of external emergencies. To induce that hospitals who are profit maximizers also maximize medical quality, reimbursement systems should make sure that these mismatches are eliminated. Here, one might consider decreasing the monetary costs of rejecting external emergency patients and especially elective surgery patients, while the monetary costs of early discharging patients or rejecting internal emergency patients might be increased.

The results of our approach vary if different parameters are selected. As mentioned in the previous section, we provide a detailed sensitivity analysis based on different parameter combinations (Appendix E.1) and parameter misspecification (Appendix E.2) in the online appendix.

## Scenario analysis

The admission and discharge policies discussed in the previous section were based on two sets of cost estimations for our case hospital. In this section, we take a broader look by varying capacities and costs in simulation (1000 simulation runs each). We consider two different variations of scenarios: First, we derive strategic implications by changing the available number of ICU resources: What are the benefits (costs) of adding (removing) one additional bed? Second, we drop the assumption of either considering purely the medical or the monetary perspective, and allow combinations of both. Based on 20 costs settings consisting of linear combinations of medical and monetary costs, we derive an efficiency frontier. In the online appendix, we consider two more variations. In Appendix B.1, we consider different cost parameter settings for the medical costs by increasing or decreasing each cost parameter by 50%, leading to 32 additional scenarios. Thus, we can demonstrate the value of our model based on different cost settings. In Appendix B.2, we analyze the impact of estimation errors on performance by comparing policies based on those 32 additional cost scenarios, while the costs of our case studies represent the ground truth. This sensitivity analysis demonstrates the cases where our model performance is robust, and those where wrong cost estimations lead to severe consequences. Please note that the appendix relates to classical sensitivity analyses. As high uncertainty is typically associated with the estimation of medical consequences, we concentrate on medical costs in these analyses. We do not perform a dedicated sensitivity analysis for the arrival rates, as this is to some extend already captured by the variation of ICU capacity.

### Strategic implications

Besides calculating optimal admission and discharge policies, the model can also serve to obtain insights on a strategic level, for example, regarding the dimensioning of ICU capacity. The model computes admission and discharge policies to minimize costs (that could be medical or monetary) based on a given capacity. Thus, by varying this capacity, we can estimate the benefits or costs of capacity changes. To this end, we run our model to determine the optimal policies for both the medical and the monetary perspective for capacities of 30 to 40 beds, and apply the simulation to report the medical (increase in mortality rate) and the monetary (lost profits) results in Appendix C. The impact of capacity changes is quite linear. If the medical perspective is optimized, decreasing the capacity to 30 beds leads to an additional mortality of 700pp – meaning that, on average, 7 more patients die due to capacity shortages. In case the monetary performance is optimized, the additional mortality increases to 1128pp, resulting in more than 11 additional mortalities. The additional monetary opportunity losses to the hospital due to a decrease from 35 to 30 beds are about 0.6 million Euros (monetary perspective optimized) up to 1.3 million Euros (medical perspective optimized). Consistent with these results, the utilization is slightly increased from 94.7% to 94.9% in the medical setting, and increased from 97.4% to 98.2% in the monetary setting. Increasing the capacity from 35 to 40 beds leads to improvements: The number of additional lives saved ranges from 6.1 (medical perspective optimized) to 9.7 (monetary perspective optimized), and the reduction of monetary opportunity losses ranges from 0.4 million € (monetary perspective optimized) to 1.7 million € (medical perspective optimized). The utilization drops from 94.7% to 93.8% in the medical setting and from 97.4% to 95.8% in the monetary setting. It is interesting to see that the positive effects seem to be stronger for the perspective that is not optimized – in cases with fewer capacity shortages, the trade-off between medical and monetary consequences seems to disappear. However, fixed costs clearly increase with capacity. Besides costs for new equipment, different (non-continuous) staffing requirements must be considered.

**Fig. 10:**
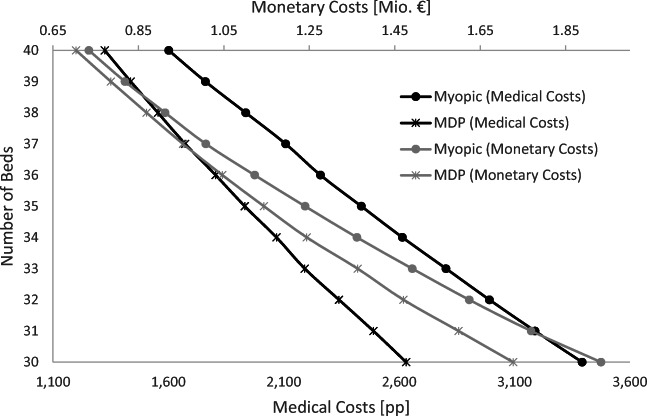
The capacity saved by MDP

Furthermore, we can also change the point of view and ask how many beds we can save by switching from myopic to MDP policies (Fig. 10). More precisely, the same cost level can be obtained with fewer beds. Especially when focusing on the medical perspective, the MDP saves between two and five beds compared to the myopic policy at the same cost level. For instance, implementing the myopic policy in the ICU with 34 beds results in an expected mortality due to capacity shortages of around 2600pp The same figure is achieved when implementing the MDP policy with a capacity of only 30 beds. Focusing on monetary goals, the differences are less pronounced. Thus, the approach may help to provide valuable input when making capacity dimensioning decisions. Besides strategic level planning, when making operational decisions, such as closing beds in the ICU because of staff shortages, it can help as well to show the resulting consequences in both medical and monetary perspectives.

### Trade-off between medical and monetary costs

**Fig. 11:**
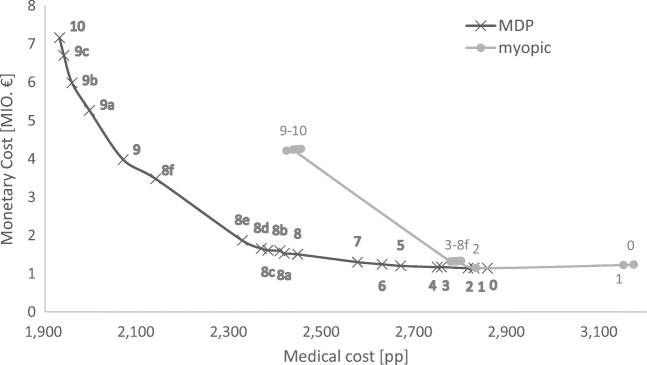
The trade off between medical and monetary cost

The case study in Section 5 demonstrated that different cost perspectives lead to different policies. While we previously focused on either the medical or the monetary perspective, we now honor the fact that these are two extreme cases. In reality, this is a problem with two objectives which are both simultaneously important to the decision maker. Thus, we now numerically construct an efficient frontier (line with “x” in Fig. 11) that contains policies which are optimal for a certain weighted combination of the medical and monetary perspectives. Note that in order to have comparable figures, we rescale monetary costs by a factor of 1/1,000 in this analysis. All points to the right/above the frontier are inefficient because at least one perspective can be improved without worsening the other. As our cost function is linear, this frontier can be easily constructed by considering convex combinations of the two cost perspectives’ parameters. To obtain points on this frontier, we first calculated 11 cases with different weightings of the two perspectives by increasing the relative weight for medical costs from 0 (case 0) to 1 (case 10) in ten steps of 0.1. In order to capture all parts of the efficient frontier, we additionally inserted 10 non-equidistant cases between the aforementioned (e.g. case 8a with a weight of 0.825 for medical costs). For each case, the resulting optimal policy is evaluated in simulations as before, and medical as well as monetary costs are recorded. The policy is quite insensitive to the weights in some areas (e.g. cases 0, 1, and 2 with relative weights of medical costs of 0 to 0.2), resulting in very similar cost values for these cases. In other areas, a small change in weights (e.g. cases 8e and 8f with relative weights for medical costs of 0.8915 and 0.8916, respectively) results in a change in the policy with big effects on costs. More information and resulting policies are given in Appendix D.

As its weight increases from case 0 to case 10, the resulting total medical costs decrease from 2855pp to 1931pp, which means that, on average, 9.2 patients die less due to capacity shortages. The reverse is also true: If we put stronger emphasis on the monetary perspective, monetary costs decrease, while mortality increases. As usual, this trade-off is not linear. When starting to increase the weight on medical costs in the first nine cases, the medical perspective can be improved at relatively low monetary costs: By decreasing the medical costs from 2,855pp to 2445pp (saving around four patients, that is, 44% of the potential decrease of medical costs) the monetary costs only increase from 1.14 to 1.51 million € (costing around 370,000 €, that is, 6% of the potential increase of monetary costs). After case 9, improving the medical perspective gets more expensive – now, the policies start to reject external emergencies and scheduled surgery patients. Switching from case 9 to case 10 will save in expectation one life (decrease of medical costs from 2069pp to 1931pp), and will lead to a monetary cost increase of 3.2 million € (increase of monetary costs from 3.98 million € to 7.16 million €).

In addition to the MDP policies, Fig. 11 also contains the results of the myopic policies (line with circles). These policies myopically decide on weighted costs. Obviously, the policy rarely changes as the weights vary. Here, the policies only change if the order of costs change. When we compare the performance of the MDP and the myopic policy in all 20 cases, the potential of the MDP solution becomes obvious: Considering case 9 with a myopic policy as a benchmark (remember that the myopic policy resembles policies used in practice), we could either reduce mortality by around four patients (from 2453pp to 2069pp), combined with a slight reduction of costs by moving to case 9 with the MDP solution, or reduce costs by 2.75 million € (from 4.26 to 1.51 million €), keeping the medical costs constant, by moving to case 8 with the MDP solution. We draft the resulting policies in Appendix D. The most noticable change is that the MDP starts to reserve beds by deferring external emergencies (starting from case 2), and by canceling scheduled surgeries (especially in case 8a, where the high monetary costs of such policies are not considered) when moving from monetary- to medical-oriented policies.

According to the sensitivity analysis based on different parameter combinations (Appendix E.1), our model has significant benefits in most of the considered test cases, there are a few cases where the MDP does not reserve beds, and its use does not lead to a considerable improvement compared to a myopic policy. The sensitivity analysis of parameter misspecification (Appendix E.2) shows that erroneous estimation of cost parameters may indeed lead to dramatic results. The worst impact on medical costs was observed for combinations of overestimation of rejection costs and underestimation of the cost of early discharges, while results are otherwise relatively robust.

## Conclusion and future research

Congestion problems in ICUs lead to dramatic negative effects on patients’ health. Both rejections of arriving patients and early discharges of existing patients lead to worse outcomes. This paper proposes a method to define admission and early discharge policies that minimize these negative consequences. Our approach applies a discrete-time Markov decision process that is solved to optimality for realistic instances. We demonstrate that by minimizing the medical consequences, the approach significantly outperforms a myopic policy as applied by most hospitals in practice. Besides, we demonstrate that different objectives lead to different policies. If, for example, monetary profits are optimized, the medical outcome is strongly affected. We extend this logic to develop an efficiency frontier covering medical and monetary perspectives, and thereby contribute to the ongoing discussion on the trade-off between medical quality and monetary costs. We further provide robustness checks and situations in medical perspective where our approach is sensitive to cost changes and to cost estimation errors. Our model provides particularly high potential in cases with low medical costs for rejecting external emergencies and high costs for early discharging low-severity patients. It is relatively robust against underestimation of rejection costs for scheduled surgeries and external emergencies and overestimation of early discharge cost of high-severity patients. However, the opposite case, that is, overestimation of rejection costs and underestimation of early discharge costs, leads to inferior results.

Various applications of our approach exist. The major one is a framework to develop recommendations for admission and discharge control on a tactical decision level. One could, for example, use it to develop simple guidelines. Such decision rules may have the form that if a certain number of high-severity patients are treated in the ICU, no more elective surgeries will be scheduled that require postoperative ICU treatment, or define occupancy levels where coordinating units are informed to divert ambulances with external emergencies to other hospitals. The policies as we illustrate them could be printed out and the ICU manager could have them as a poster in the ICU – no additional information systems would be required. An additional application is to use the approach as decision support for capacity dimensioning on strategic and operational decision level. It provides insights on the consequences of capacity shortages, and allows decision makers to consider different objectives within the admission and discharge policies. In both cases, our approach has direct managerial applications.

We believe that managing ICU admissions and discharges is of great importance, and has large potential for future research. To focus on the trade-off between medical and monetary goals, and to allow easy implementation of our proposed policies in practice, we aggregated situations (e.g. day and night shift) and patient types. From a modeling point of view, adding a higher level of complexity could be of interest – even though this might reduce the ease of implementation. Possible extensions include a time-dependent arrival and discharge process (e.g., discharge at specific time of the day, arrival rates vary on different time slots and weekday), a more detailed clustering of patient types (e.g., cluster patients according to the specific symptoms and objective criteria), and the modelling of re-admissions or delayed admissions of rejected or discharged patients. These extensions may lead to a more complex model that cannot be solved to optimality. Thus, approximation schemes such as approximate dynamic programming may be necessary. Another approach may be to consider variations of the setting. For example, our results have shown that in a full ICU, there is usually at least one low-severity patient. If the medical perspective is optimized, there are at least 3 low-severity patients with a probability of about 85%, while this figure is lower for the monetary perspective. Thus, if inferior beds with a lower level of care for low-severity patients are considerably cheaper, a multi-tiered ICU should be considered. Last but not the least, the control policies implemented in the ICU might influence the other departments as well, and the interdependencies of the ICU on the rest of the hospital is an interesting topic to study.

## Supplementary Information

ESM 1 (DOCX 1.34 mb)

## References

[1] Hyer NL, Wemmerlöv U, Morris JA (2009). Performance analysis of a focused hospital unit: The case of an integrated trauma center. J. Oper. Manag..

[2] Coopersmith CM, Wunsch H, Fink MP, Linde-Zwirble WT, Olsen KM, Sommers MS, Anand KJS, Tchorz KM, Angus DC, Deutschman CS (2012). A comparison of critical care research funding and the financial burden of critical illness in the United States. Crit Care Med.

[3] Halpern NA, Pastores SM (2010) Critical care medicine in the United States 2000-2005: An analysis of bed numbers, occupancy rates, payer mix, and costs. Crit Care Med 38(1):65–71 10.1097/CCM.0b013e3181b090d0 19730257

[4] Thompson S, Nunez M, Garfinkel R, Dean MD (2009) OR practice - efficient short-term allocation and reallocation of patients to floors of a hospital during demand surges. *Oper. Res.* 57(2):261–273

[5] Boyd O, Evans L (2016). The future workforce of our intensive care units – doctor, physician assistant or no-one?. J Intensive Care Soc.

[6] Bittencourt O, Verter V, Yalovsky M (2018). Hospital capacity management based on the queueing theory. Int J Product Perform Manag.

[7] Bai J, Fügener A, Schoenfelder J, Brunner JO (2018) Operations research in intensive care unit management: A literature review. Health Care Manag. Sci. 21(1):1–24 10.1007/s10729-016-9375-1 27518713

[8] Zimmerman JE, Kramer AA, McNair DS, Malila FM, Shaffer VL (2006) Intensive care unit length of stay: Benchmarking based on acute physiology and chronic health evaluation (APACHE) IV. *Crit. Care Med.* 34(10):2517–2529 10.1097/01.CCM.0000240233.01711.D9 16932234

[9] Cardoso LTQ et al (2011) Impact of delayed admission to intensive care units on mortality of critically ill patients: A cohort study. *Crit. Care* 15(1):R28 10.1186/cc9975 PMC3222064 21244671

[10] Hung S-C (2014). Determining delayed admission to intensive care unit for mechanically ventilated patients in the emergency department. Crit. Care.

[11] Chan CW, Farias VF, Escobar GJ (2016). The impact of delays on service times in the intensive care unit. Manag Sci.

[12] Chan CW, Farias VF, Bambos N, Escobar GJ (2012). Optimizing intensive care unit discharge decisions with patient readmissions. Oper. Res..

[13] Dobson G, Hasija S, Pinker EJ (2011). Reserving capacity for urgent patients in primary care. Prod Oper Manag.

[14] Li X, Liu D, Geng N, Xie X (2019). Optimal ICU admission control with premature discharge. IEEE Trans Autom Sci Eng.

[15] Diwas Singh KC, Terwiesch C (2012). An econometric analysis of patient flows in the cardiac intensive care unit. Manuf Serv Oper Manag.

[16] Bai J, Gerstmeyr S, Brunner JO (2020) Simulation and evaluation of icu management policies, in Proceedings of the 2020 winter simulation conference

[17] Kim SC, Horowitz I (2002) Scheduling hospital services: The efficacy of elective-surgery quotas. Omega 30:335–346

[18] Litvak N, van Rijsbergen M, Boucherie RJ, van Houdenhoven M (2008). Managing the overflow of intensive care patients. Eur. J. Oper. Res..

[19] Yang M (2013). A model to create an efficient and equitable admission policy for patients arriving to the cardiothoracic ICU. Crit. Care Med..

[20] Nates JL et al. (2016) ICU admission, discharge, and triage guidelines, 44, (8) 10.1097/CCM.0000000000001856 27428118

[21] Fügener A, Hans EW, Kolisch R, Kortbeek N, Vanberkel PT (2014). Master surgery scheduling with consideration of multiple downstream units. Eur J Oper Res.

[22] Fügener A, Schiffels S, Kolisch R (2017) Overutilization and underutilization of operating rooms - Insights from behavioral health care operations management. Health Care Manag Sci 20(1):115–128 10.1007/s10729-015-9343-1 26433372

[23] Pearse RM, Moreno RP, Bauer P, Pelosi P, Metnitz P, Spies C, Vallet B, Vincent JL, Hoeft A, Rhodes A (2012). Mortality after surgery in Europe: a 7 day cohort study. Lancet (London, England).

[24] Kagerbauer S, Blobner M, Ulm B, Jungwirth B (2020) Wie maschinelles Lernen Anästhesie und Intensivmedizin prägt. Clin Anaesth 61:85–96

[25] Jauk S, Kramer D, Stark G, Hasiba K, Leodolter W, Schulz S, Kainz J (2019). Development of a machine learning model predicting an ICU admission for patients with elective surgery and its prospective validation in clinical practice. Stud Health Technol Inform.

[26] Chalfin DB, Trzeciak S, Likourezos A, Baumann BM, Dellinger RP (2007). Impact of delayed transfer of critically ill patients from the emergency department to the intensive care unit. Crit. Care Med..

[27] Durbin CJ, Kopel R (1993). A case-control study of patients readmitted to the intensive care unit. Crit. Care Med..

[28] Chen L, Martin C, Keenan S, Sibbald W (1998). Patients readmitted to the intensive care unit during the same hospitalization : Clinical features and outcomes. Crit. Care Med..

[29] Chalfin D (2005). Length of intensive care unit stay and patient outcome: The long and short of it all. Crit. Care Med..

[30] Helm JE, Alaeddini A, Stauffer JM, Bretthauer KM, Skolarus TA (2016). Reducing hospital readmissions by integrating empirical prediction with resource optimization. Prod. Oper. Manag..

[31] Chrusch CA, Olafson KP, McMillan PM, Roberts DE, Gray PR (2009). High occupancy increases the risk of early death or readmission after transfer from intensive care. Crit. Care Med..

[32] G. Iapichino *et al.*, Volume of activity and occupancy rate in intensive care units. Association with mortality, *Intensive Care Med.*, vol. 30, no. 2, pp. 290–297, 2004 10.1007/s00134-003-2113-4 14685662

[33] Bouneb R, Mellouli M, Dardouri M, Ben Soltane H, Chouchene I, Boussarsar M (2018). Determinants and outcomes associated with decisions to deny intensive care unit admission in Tunisian ICU. Pan Afr Med J.

[34] Louriz M, Abidi K, Akkaoui M, Madani N, Chater K, Belayachi J, Dendane T, Zeggwagh AA, Abouqal R (2012). Determinants and outcomes associated with decisions to deny or to delay intensive care unit admission in Morocco. Intensive Care Med.

[35] Kim SH, Chan CW, Olivares M, Escobar G (2015). ICU admission control: an empirical study of capacity allocation and its implication on patient outcomes. Manag Sci.

[36] Shmueli A, Sprung CL, Kaplan EH (2003). Optimizing admissions to an intensive care unit. Health Care Manag. Sci..

[37] Hu W, Chan CW, Zubizarreta JR, Escobar GJ (2018). An examination of early transfers to the ICU based on a physiologic risk score. Manuf Serv Oper Manag.

[38] Long EF, Mathews KS (2018). The boarding patient: effects of ICU and hospital occupancy surges on patient flow. Prod Oper Manag.

[39] Miedaner F, Sülz S (2019) Boundaries of focus and volume: An empirical study in neonatal intensive care. Prod Oper Manag 0(0):1–11

[40] Griffiths JD, Price-Lloyd N, Smithies M, Williams J (2006). A queueing model of activities in an intensive care unit. IMA J Manag Math.

[41] Kim SC, Horowitz I, Young KK, Buckley TA (1999). Analysis of capacity management of the intensive care unit in a hospital. Eur. J. Oper. Res..

[42] Chan C, Yom-Tov G (2011) Intensive care unit patient flow with readmissions : A state-dependent queueing network, in 2011 MSOM Annual Conference Ann Arbor, Michigan

[43] Barz C, Rajaram K (2015). Elective patient admission and scheduling under multiple resource constraints. Prod Oper Manag.

[44] Samiedaluie S, Kucukyazici B, Verter V, Zhang D (2017). Managing patient admissions in a neurology ward. Oper Res.

[45] Zonderland ME, Boucherie RJ, Litvak N, Vleggeert-Lankamp CLAM (2010). Planning and scheduling of semi-urgent surgeries. Health Care Manag. Sci..

[46] Yang M, Fry MJ, Scurlock C (2015). The ICU will see you now: efficient-equitable admission control policies for a surgical ICU with batch arrivals. IIE Trans.

[47] Gocgun Y, Puterman ML (2014). Dynamic scheduling with due dates and time windows: An application to chemotherapy patient appointment booking. Health Care Manag. Sci..

[48] Gupta D, Wang L (2008). Revenue Management for a Primary-Care Clinic in the presence of patient choice. Oper Res.

[49] Yu S, Kulkarni VG, Deshpande V (2019). Appointment scheduling for a health care facility with series patients. Prod Oper Manag.

[50] Li D, Ding L, Connor S (2019). When to switch? Index policies for resource scheduling in emergency response. Prod Oper Manag.

[51] Xie AJ et al. (2020) Analytics for hospital resource planning — two case studies, POMS

[52] Dobson G, Lee H-H, Pinker E (2010). A model of ICU bumping. Oper Res.

[53] Li J, Dong M, Zhao W (2015). Admissions optimisation and premature discharge decisions in intensive care units. Int J Prod Res.

[54] Dobson G, Lee HH, Pinker E (2010). A model of ICU bumping. Oper. Res..

[55] Verburg IWM, De Keizer NF, De Jonge E, Peek N (2014) Comparison of regression methods for modeling intensive care length of stay, PLoS One, 9 (10) 10.1371/journal.pone.0109684 PMC4215850 25360612

[56] Sprung CL (1999). Evaluation of triage decisions for intensive care admission. Crit. Care Med..

[57] Azoulay E (2001). Compliance with triage to intensive care recommendations. Crit. Care Med..

[58] Joynt G, Gomersall C, Tan P, Lee A, Cheng C, Wong E (2001). Prospective evaluation of patients refused admission to an intensive care unit: Triage, futility and outcome. Intensive Care Med..

[59] McManus ML, Long MC, Cooper A, Litvak E (2004). Queuing theory accurately models the need for critical care resources. Anesthesiology.

