# Supplementary material for: Managing admission and discharge processes in intensive care units
Source: Health Care Manag Sci. 2021 Jun 10;24(4):666–85. doi: 10.1007/s10729-021-09560-6 (PMC8189840; doi:10.1007/s10729-021-09560-6)
Supplement: Supplementary file 1 — (DOCX 1.34 mb) [file 10729_2021_9560_MOESM1_ESM.docx]

**Online Appendix**

**Managing admission and discharge processes in intensive care units**

Appendix A: Solution Approach

The standard approach to compute a Markov decision process is to simply solve the recursive value function backwards in time. That is, starting with $T,$ $V_{t}\left( \boldsymbol{S}_{t} \right)$ is solved for all $\boldsymbol{S}_{t}$ using the previously calculated values of $V_{t+1}\left( \boldsymbol{S}_{t+1} \right)$. This process may suffer from 3 curses of dimensionality (see, for example, Powell [1] Chapter 1): the size of the state space, the size of the action space, and the computation of the expectation.

- The size of the state space is $(\frac{\left( B+1 \right)\left( B+2 \right)}{2})\cdot4$ and, thus, grows quadratic in $B$. This is no issue, given that real-world ICUs are between 10 and 50 beds in size.
- Likewise, the action space with its three binary dimensions is limited to $2^{3}=8$ actions.
- The calculation of the expectation is a bit more complicated, as it depends on ICU occupancy. The number of possible state transitions (possible realizations of $\boldsymbol{\omega}_{t+1}$) is bounded by $4\cdot\frac{B^{2}}{2}\cdot\frac{B^{2}}{2}\cdot2=2B^{4}$. Note that the number of state transitions can be considerably reduced by neglecting transitions with very low probabilities (like 10 patients out of 10 improving from high-severity to low-severity in one period).

More specifically, a pseudo code for the standard backward dynamic programming algorithm is as follows:

**Step 0**. Initialize the terminal contributions $V_{T+1}\left( \boldsymbol{S}_{t+1} \right) \forall\boldsymbol{S}_{t+1}$, set $t=T$

**Step 1**. Calculate $V_{t}\left( \boldsymbol{S}_{t} \right)=\min_{\boldsymbol{a}_{t}} \left\{ C\left( \boldsymbol{S}_{t},\boldsymbol{a}_{t} \right)+\mathbb{E}_{\boldsymbol{\omega}_{t}}\left[ V_{t+1}\left( \boldsymbol{S}_{t+1}\left( \boldsymbol{S}_{t},\boldsymbol{a}_{t},\boldsymbol{\omega}_{t+1} \right) \right) \right] \right\} \forall\boldsymbol{S}_{t}$.

**Step 2**. If $t>0$**,** decrement $t$ and go to step 1. Else stop.

Appendix B: Estimation of cost parameters

B.1. Medical perspective

From a medical point of view, we chose the absolute increase of mortality rates as the single performance indicator and cost component. Please note that the following numbers are percentage points (pp), that is, differences of percentages. If, for example, due to an action the mortality rate of a patient increases from 10% to 11%, we denote the increase as 1pp. In this paragraph, we discuss changes in mortality rates due to the five possible actions mentioned above and state the assumed parameter values for our case study. After obtaining ranges from the literature, we discussed the concrete cost parameter with the ICU manager of the case hospital, and selected the values that fit best for our case hospital. However, those parameters contain some uncertainty. Thus, we discuss the impact of different parameter choices and of estimation errors of those parameters later in this online appendix.

- $c_{1, med}^{rej}=1\mathrm{pp}$. Rejection of an *elective surgery patient* typically results in rescheduling, that is, delaying the surgery, or scheduling it at another hospital. There is little literature on the medical consequences of cancelling surgeries in general. However, for certain orthopedic surgeries, there is data on mortality rates available. A widely investigated example is hip fracture surgeries, where some studies find no significant effect of delays on mortality rates [2], while others (for example, Shiga et al. [3]) detect systematic increases of mortality rates. Shiga et al. [3] performed a meta-analysis on hip fracture reports. They report an average short-term mortality rate of 7% for non-delayed surgeries, which increases to 10% for surgeries being delayed. Nyholm et al. [4] show that delaying surgeries of proximal femoral fracture leads to increases of mortality rates between 1pp and 4pp. Thus, the increase of mortality rate due to delays reported in the literature covers a range between 0pp and 4pp. Cancer surgeries with a high risk of complications, such as esophagectomy, whipple procedure, or cystectomy are changing rapidly due to new surgical approaches, improved surgical training, and oncological supportive treatment (Sabra et al. [5], Yibulayin et al. [6]). Therefore, we decided to use data of an index surgical procedure with stable mortality and ICU admission rates, the hip and the proximal femoral fracture, of which the surgical technique and the affected population did not change as much over the last decade. While orthopedic procedures that are often performed with old patients rather provide an upper bound of negative consequences of cancelling scheduled surgeries, they do account for a meaningful share of cases that are relevant to ICU management. In our case study, we assume an absolute increase of the mortality rate of 1pp due to rejection of an elective surgery patient.
- $c_{2, med}^{rej}=15\mathrm{pp}$*. Internal emergencies* cover patients who are treated within the hospital when their medical condition unexpectedly deteriorates. They could be located at a regular ward, an operating theater, or an emergency department. Rejecting these patients at the ICU typically means they need to be treated within a regular ward using extra nursing capacities. The studies of Kim et al. [7] and Kime et al. [8] claim that rejecting these patients increases mortality rates by more than 20pp from around 30% to more than 50%. Checkley [9] and Iapichino et al. [10] report that internal emergency patients whose admission is initially denied show mortality rates that are 10pp above those who are directly admitted (Checkley [9] reports 27% mortality for admitted versus 37% for denied admission, Iapichino et al. [10] 28% versus 39%). In our case study, we assume an absolute increase of the mortality rate of 15pp due to rejection of an internal emergency patient.
- $c_{3, med}^{rej}=3\mathrm{pp}$. Rejecting *external emergency* patients typically means informing a central coordination center that no emergency patients can be treated, so that emergency ambulances will be directly diverted to other hospitals. Emergency ambulances which have already arrived at the hospital might be sent away. In all cases, external patients will experience a delay in their treatment, which leads to an increase of mortality rates. Chalfin et al. [11] report mortality increases of 2pp to 5pp due to delayed ICU admissions (mortality in the ICU from 8.4% to 10.7%, mortality during the total stay from 12.9% to 17.4%), while Singer et al. [12] find that increased boarding times at emergency departments, which could be caused by ICU rejections, lead to an increase in mortality rates of around 2pp (mortality over all patients from 2.5% to 4.5%). In our case study, we assume an increase of the mortality rate of 3pp due to rejection of an external emergency patient.
- $c_{1, med}^{edis}=2\mathrm{pp}$. Chrusch et al. [13] assume that a congested ICU provokes *early discharges of low-severity patients*. They find a higher level of re-admissions (around 4pp higher) and higher mortality rate for re-admitted patients (21.3% against 0.3% for patients in the wards who were not re-admitted). Thus, the increase of mortality of the least critically ill patients due to readmissions was close to 1pp. Furthermore, the ICU mortality for readmitted patients is slightly higher than the one for a primary stay (21.3% against 19.0%). In our case study, we assume an absolute increase of the mortality rate of 2pp due to the early discharge of a low-severity patient.
- $c_{2, med}^{edis}=10\mathrm{pp}$. There are few studies analyzing the effects of *early discharges of high-severity patients*. Some studies consider the mortality of high-severe versus low-severe patients: Smith et al. [14] analyze the effects on mortality based on the health status at the time of discharge. They show that patients discharged with a high criticality index exhibit mortality rates that are about 18pp higher compared to patients discharged with a low criticality index (21.4% compared to 3.7%). Daly et al. [15] show that discharge of patients with high-severity leads to mortality rates of 25% compared to 4% for less risky patients. For high-severity patients, these mortality rates strongly exceed those of staying within the ICU. Chan et al. [16], for example, note that within the ICU, the difference between high-severity patients and low-severity patients is around 10pp (14.6% compared to 4.2%). Obviously, these figures cannot be matched precisely, as not all high-severity patients will become low-severity patients if they are not early discharged. In our case study, we assign an absolute increase of the mortality rate of 10pp due to the early discharge of a high-severity patient.

B.2. Monetary perspective

Rising cost pressure on hospitals increases the importance of a monetary perspective. ICUs are typically not profit centers, but decisions taken in the ICU might largely impact a hospital’s profitability. From a monetary perspective, we consider the costs in our model to be the profit loss for the hospital due to rejections or early discharges. Obviously, these numbers heavily depend on the reimbursement model of the relevant health care system. As previously stated, we use data from the German DRG (Diagnosis Related Group) system as of 2017 [17]. Since most hospital costs are largely fixed costs, we consider the lost revenues as a proxy for lost profits, and only add additional costs if appropriate. Thus, our values can be seen as upper bounds for lost profits. As in the medical perspective, these parameters need to be adapted for each specific hospital. While the exact numbers vary a lot, we believe that the relation between those values are similar among various hospitals and health systems. In this paragraph, we explain the logic we applied to obtain our cost parameters for the five possible actions. Please note that for clarity and to avoid pseudo-accuracy, we round all values to the nearest 100 €.

- $c_{1, mon}^{rej}=9,200 €.$If an *elective surgery patient* is rejected, the surgery will be cancelled or rescheduled. Thus, the hospital might either lose the profit for this patient (if rescheduled at another hospital), or for a similar patient (if rescheduled at the same hospital). The latter is because the operating theatre is typically the main bottleneck, and using another surgery slot results in scheduling one patient less. Thus, rejecting a planned surgery patient results in losing the average reimbursement of one patient with treatments in both the operating room and the ICU. According to the German DRG system, this value is around 9,200 €.
- $c_{2, mon}^{rej}=5,800 €.$Rejecting an *internal emergency patient* has several monetary implications. First, if the patient is not treated at an ICU, no extra charges for ICU treatment can be billed. Second, additional nursing capacities need to be booked in order to secure adequate treatment on a regular ward. Third, additional costs such as legal costs in case of negative incidents may occur. We neglect the latter because they are often covered by insurances. Based on the German reimbursement system and nursing costs, the opportunity costs for the first component is around 1,050 €, and the extra nursing for the expected length of stay (7.3 days) amounts to 4,720 €. Thus, the total value is around 5,800 €.
- $c_{3, mon}^{rej}=4,100 €.$Rejecting an *external emergency patient* typically leads to diverting the patient to another hospital. Thus, the revenue for this patient is lost and we set the cost parameter to the average reimbursement of patients with any ICU treatment, leading to a value of 4,100 €.
- $c_{1, mon}^{edis}=700 €.$The costs for *early discharging a low-severity* *patient* are difficult to estimate. In our hospital, most early discharged patients require some additional supervision from a nurse. Thus, we consider the costs of about one day (half of the expected length of stay of a low-severity patient) of extra nursing, resulting in a cost parameter of 700 €.
- $c_{2, mon}^{edis}=6,500 €.$The cost for *early discharging high-severity patients* are computed as follows: Similar to the rejection of internal emergency patients, extra nursing has to be paid for during the expected remaining length of stay in the ICU of the patient. Due to the considerably longer period of extra care (approximately 9.5 days, half of the expected length of stay of a high-severity patient) compared to low-severity patients, this cost parameter adds up to 6,500€. Again, we do not consider legal costs.

Appendix C: Effects of Changing Number of Beds (Section 6.1)

| Objective optimized | Medical | | | | Monetary | | | | |
| --- | --- | --- | --- | --- | --- | --- | --- | --- | --- |
| Approach | MDP | | Myopic | | | MDP | | Myopic | |
| Costs eval.  # Beds | Medical | Monetary | Medical | Monetary | | Medical | Monetary | Medical | Monetary |
| 30 | 2,630 | 8,479,086 | 3,393 | 5,596,936 | | 3,983 | 1,727,001 | 4,674 | 1,932,752 |
| 31 | 2,489 | 8,243,499 | 3,189 | 5,324,028 | | 3,748 | 1,599,217 | 4,329 | 1,769,349 |
| 32 | 2,339 | 7,994,341 | 2,991 | 5,057,786 | | 3,506 | 1,470,294 | 4,018 | 1,624,208 |
| 33 | 2,191 | 7,703,195 | 2,803 | 4,785,807 | | 3,297 | 1,363,047 | 3,730 | 1,490,802 |
| 34 | 2,068 | 7,444,602 | 2,614 | 4,501,967 | | 3,061 | 1,244,085 | 3,444 | 1,361,448 |
| 35* | 1,931 | 7,160,950 | 2,436 | 4,245,759 | | 2,855 | 1,143,772 | 3,172 | 1,239,946 |
| 36 | 1,805 | 6,859,778 | 2,259 | 3,964,459 | | 2,650 | 1,046,240 | 2,900 | 1,121,906 |
| 37 | 1,672 | 6,490,779 | 2,108 | 3,723,968 | | 2,453 | 954,235 | 2,637 | 1,007,819 |
| 38 | 1,554 | 6,124,937 | 1,935 | 3,451,939 | | 2,259 | 869,142 | 2,413 | 912,560 |
| 39 | 1,436 | 5,824,626 | 1,760 | 3,161,005 | | 2,067 | 785,368 | 2,188 | 818,636 |
| 40 | 1,324 | 5,456,733 | 1,601 | 2,895,646 | | 1,877 | 704,123 | 1,978 | 734,045 |

Table A.1 Results for different ICU sizes (* is the base case)

Appendix D: Trade-off between Medical and Monetary Costs (Section 6.2)

The cost settings of the 20 cases in the sensitivity analysis (the base cases of medical (case 10) and monetary (case 0) perspectives are included) are presented in Table A.2. To have medical and monetary costs on a comparable level, we denote monetary costs in units of thousand euros.

| Case | Weight_med | Weight_mon | $\boldsymbol{c}_{\boldsymbol{i=1}}^{\boldsymbol{rej}}$ | $\boldsymbol{c}_{\boldsymbol{i=2}}^{\boldsymbol{rej}}$ | $\boldsymbol{c}_{\boldsymbol{i=3}}^{\boldsymbol{rej}}$ | $\boldsymbol{c}_{\boldsymbol{j=1}}^{\boldsymbol{edis}}$ | $\boldsymbol{c}_{\boldsymbol{j=2}}^{\boldsymbol{edis}}$ |
| --- | --- | --- | --- | --- | --- | --- | --- |
| 0 | 0 | 1 | 9.2 | 5.8 | 4.1 | 0.7 | 6.5 |
| 1 | 0.1 | 0.9 | 8.38 | 6.72 | 3.99 | 0.83 | 6.85 |
| 2 | 0.2 | 0.8 | 7.56 | 7.64 | 3.88 | 0.96 | 7.2 |
| 3 | 0.3 | 0.7 | 6.74 | 8.56 | 3.77 | 1.09 | 7.55 |
| 4 | 0.4 | 0.6 | 5.92 | 9.48 | 3.66 | 1.22 | 7.9 |
| 5 | 0.5 | 0.5 | 5.1 | 10.4 | 3.55 | 1.35 | 8.25 |
| 6 | 0.6 | 0.4 | 4.28 | 11.32 | 3.44 | 1.48 | 8.6 |
| 7 | 0.7 | 0.3 | 3.46 | 12.24 | 3.33 | 1.61 | 8.95 |
| 8 | 0.8 | 0.2 | 2.64 | 13.16 | 3.22 | 1.74 | 9.3 |
| 8a | 0.825 | 0.175 | 2.435 | 13.39 | 3.1925 | 1.7725 | 9.3875 |
| 8b | 0.85 | 0.15 | 2.23 | 13.62 | 3.165 | 1.805 | 9.475 |
| 8c | 0.875 | 0.125 | 2.205 | 13.85 | 3.1375 | 1.8375 | 9.5625 |
| 8d | 0.89 | 0.11 | 1.902 | 13.988 | 3.121 | 1.857 | 9.615 |
| 8e | 0.8915 | 0.1085 | 1.8897 | 14.0018 | 3.11935 | 1.85895 | 9.62025 |
| 8f | 0.8916 | 0.1084 | 1.88888 | 14.00272 | 3.11924 | 1.85908 | 9.6206 |
| 9 | 0.9 | 0.1 | 1.82 | 14.08 | 3.11 | 1.87 | 9.65 |
| 9a | 0.925 | 0.075 | 1.615 | 14.31 | 3.0825 | 1.9025 | 9.7375 |
| 9b | 0.95 | 0.05 | 1.41 | 14.54 | 3.055 | 1.935 | 9.825 |
| 9c | 0.975 | 0.025 | 1.205 | 14.77 | 3.0275 | 1.9675 | 9.9125 |
| 10 | 1.0 | 0 | 1 | 15 | 3 | 2 | 10 |

Table A.2 Weighted cost settings in sensitivity analysis

In the following, we document the myopic and MDP policies of the cases with the weight of medical costs ranging between 0 and 1 using steps of 0.1. This corresponds to cases zero to ten of Table A.2.

| Case 0 (weight medical costs: 0) |  |
| --- | --- |
|  |  |

| Case 1 (weight medical costs: 0.1) | 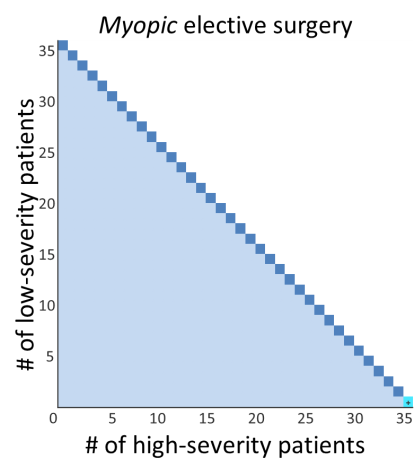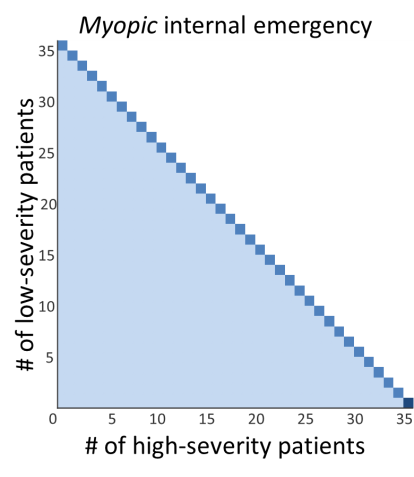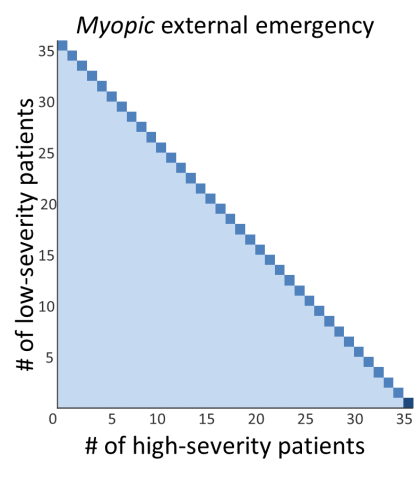 |
| --- | --- |
|  | 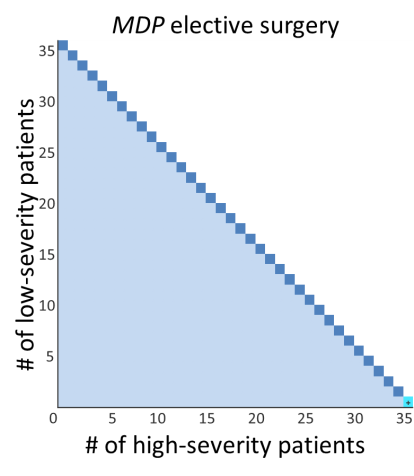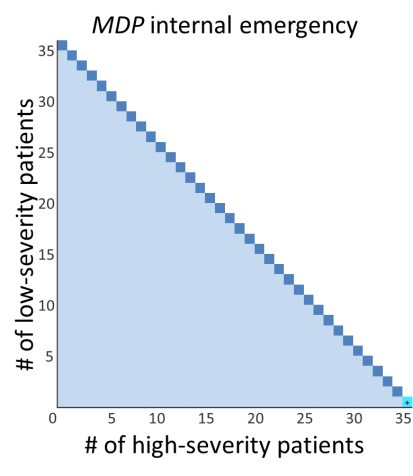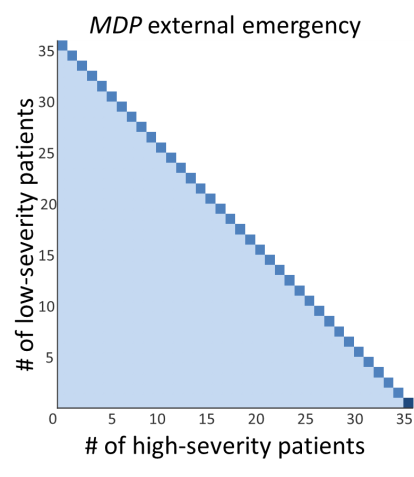 |
| Case 2 (weight medical costs: 0.2) | 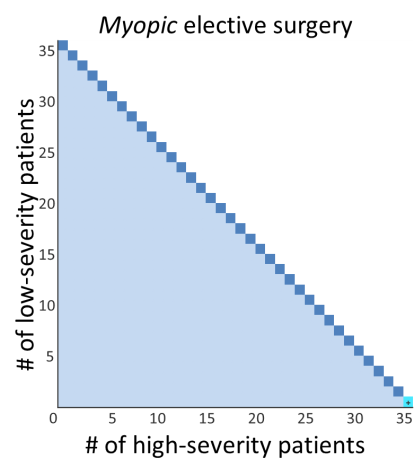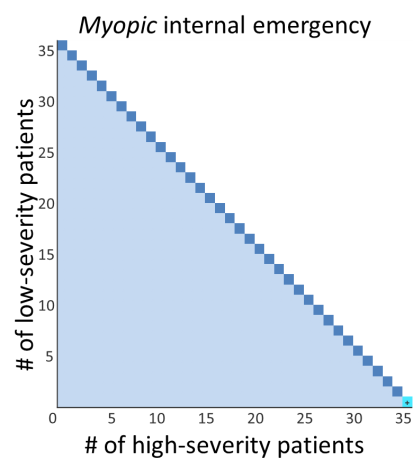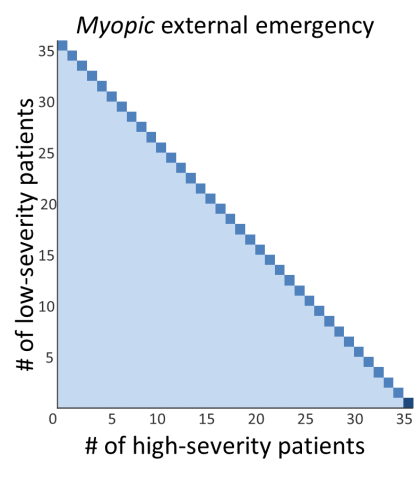 |
|  | 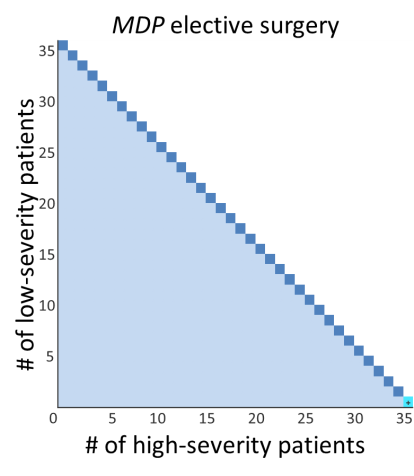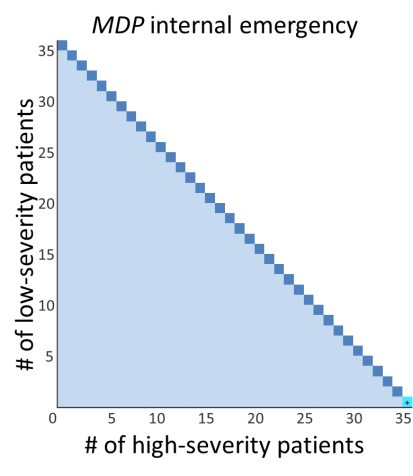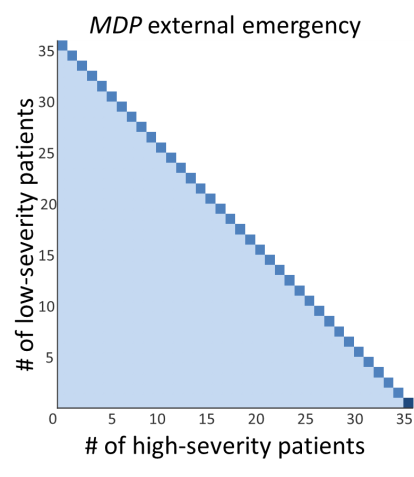 |

| Case 3 and 4 (weight medical costs: 0.3 and 0.4) | 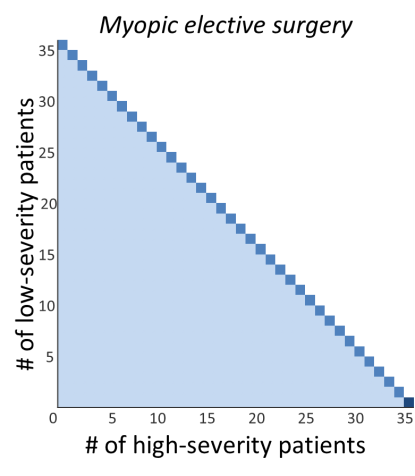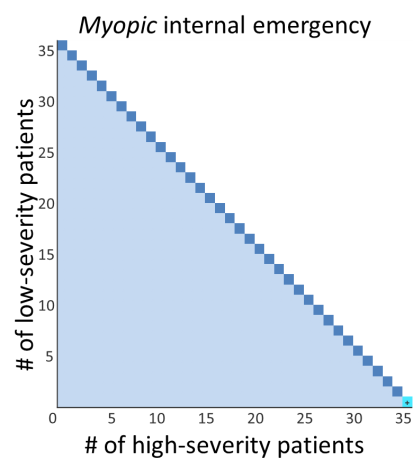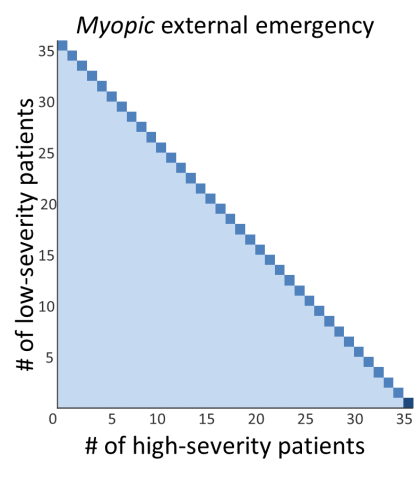 |
| --- | --- |
|  | 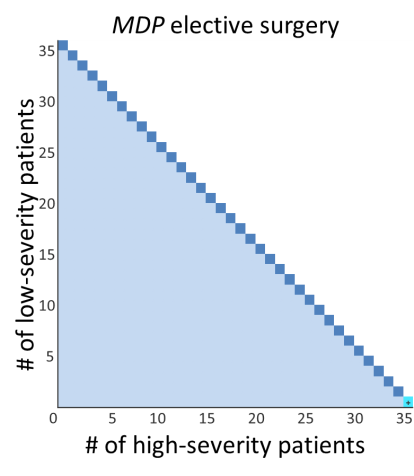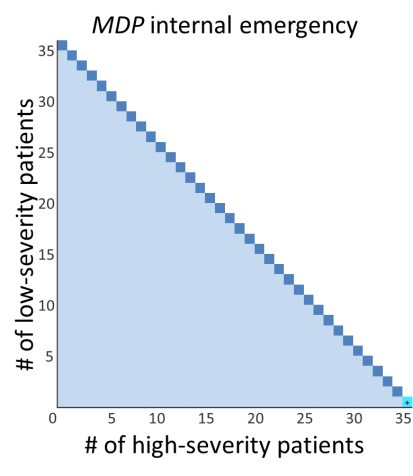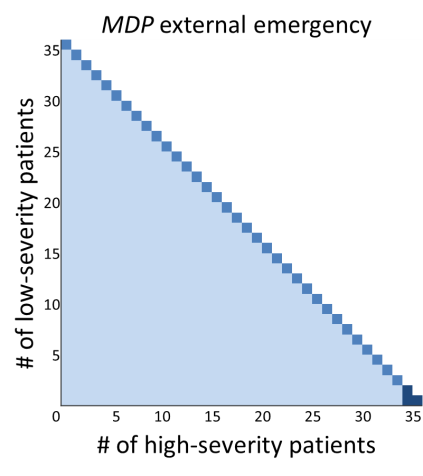 |
| Case 5 (weight medical costs: 0.5) | 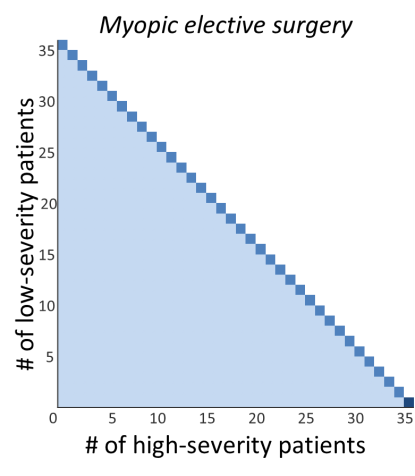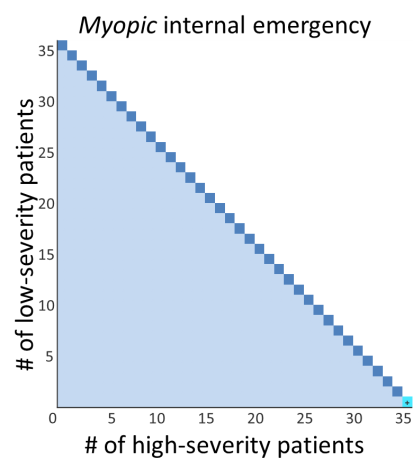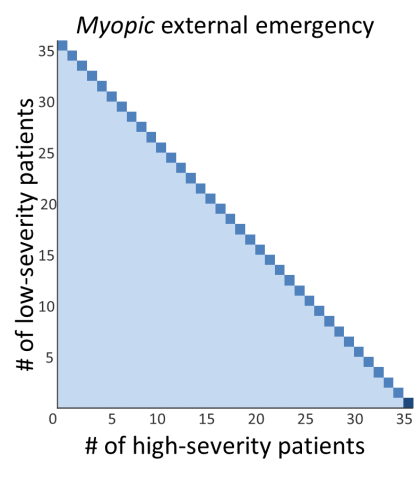 |
|  | 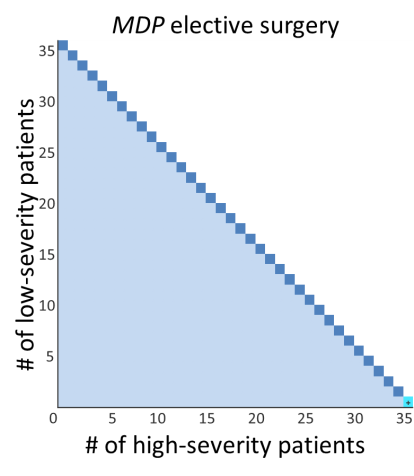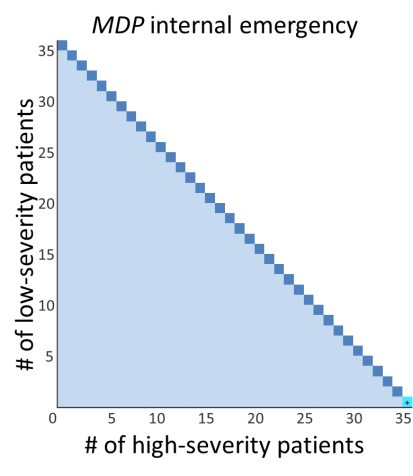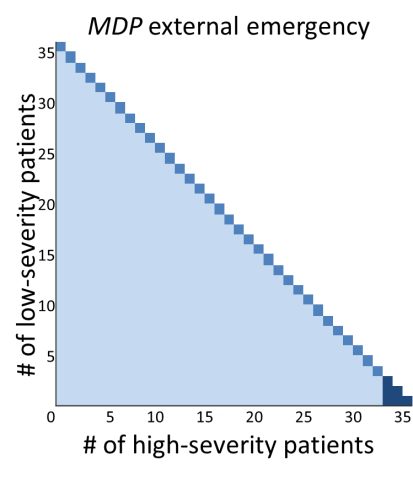 |

| Case 6 (weight medical costs: 0.6) | 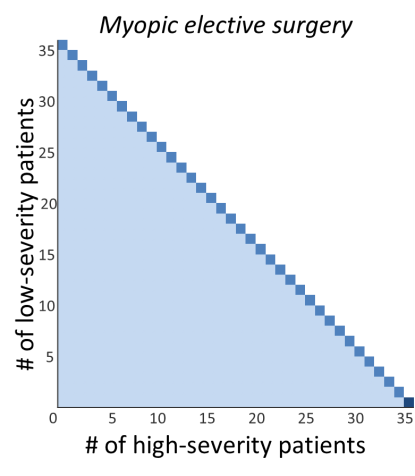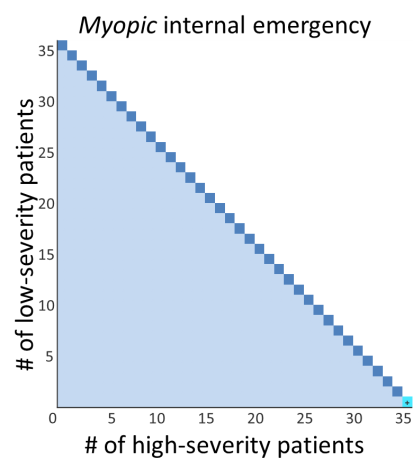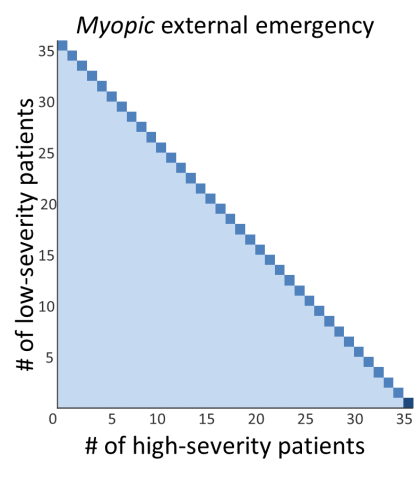 |
| --- | --- |
|  | 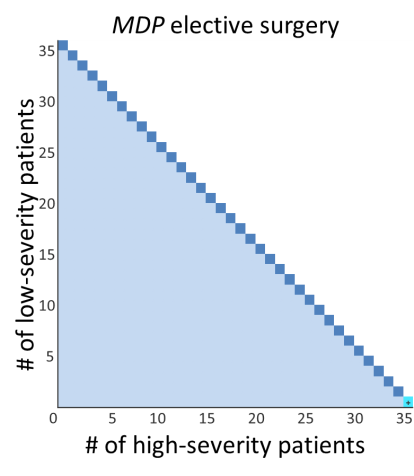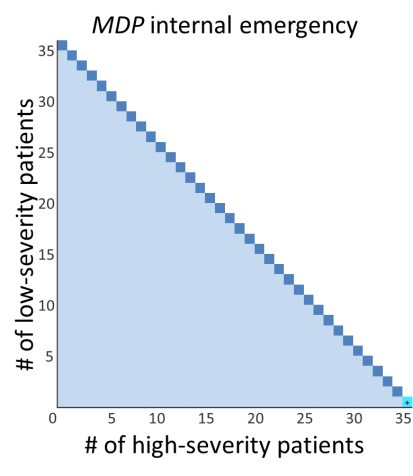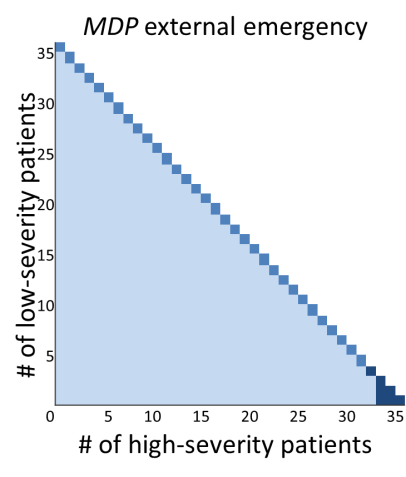 |
| Case 7 (weight medical costs: 0.7) | 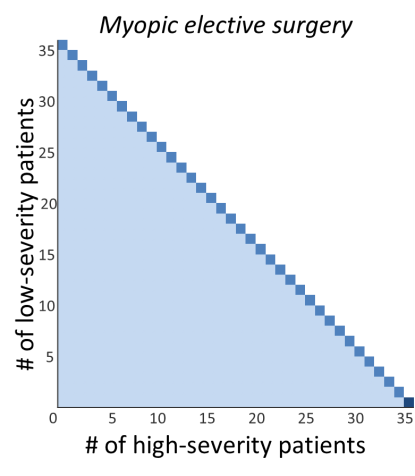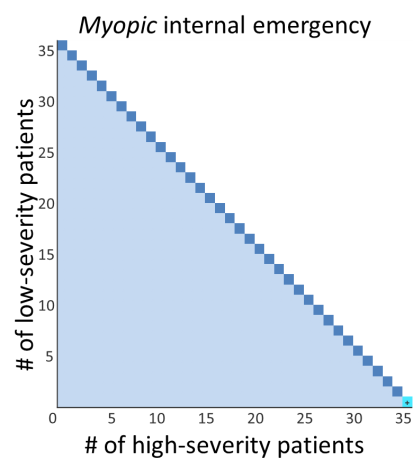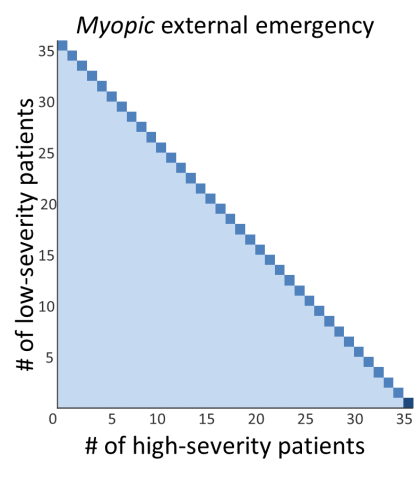 |
|  | 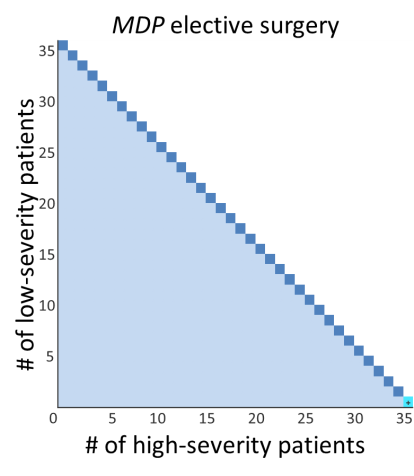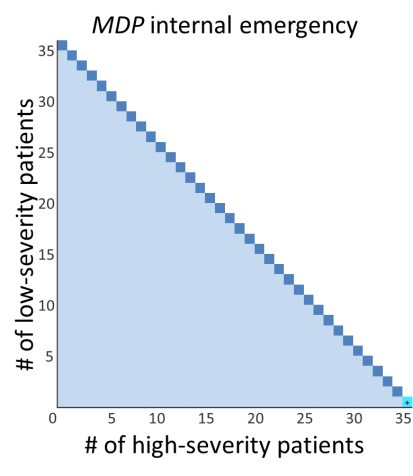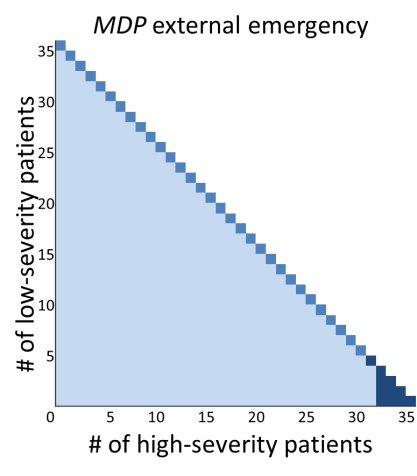 |

| Case 8 (weight medical costs: 0.8) | 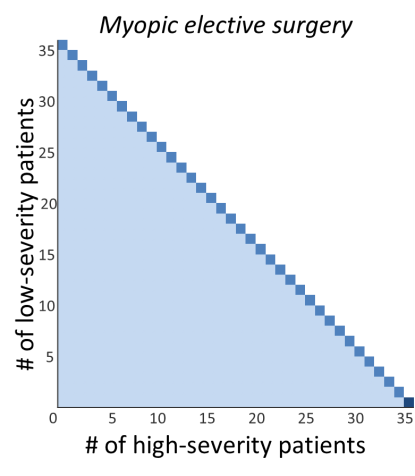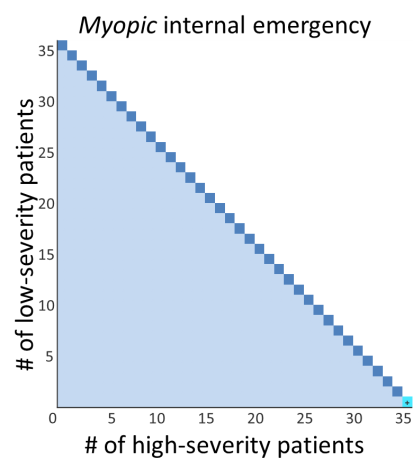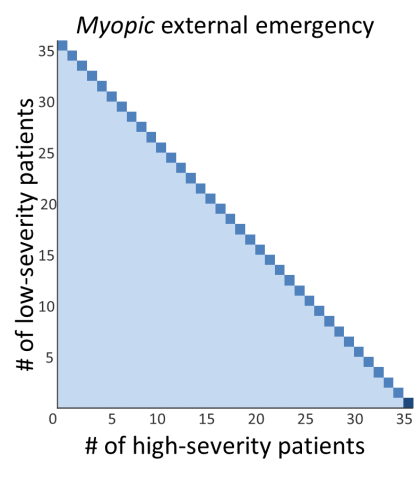 |
| --- | --- |
|  | 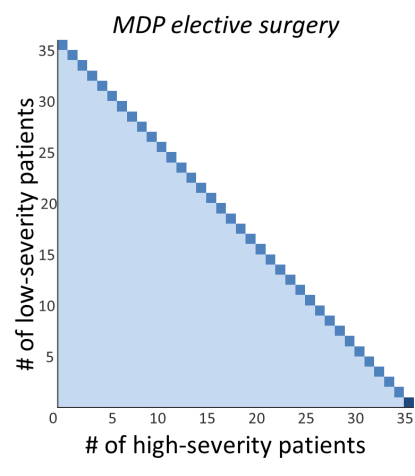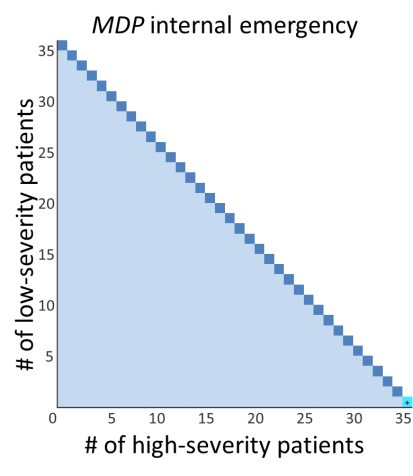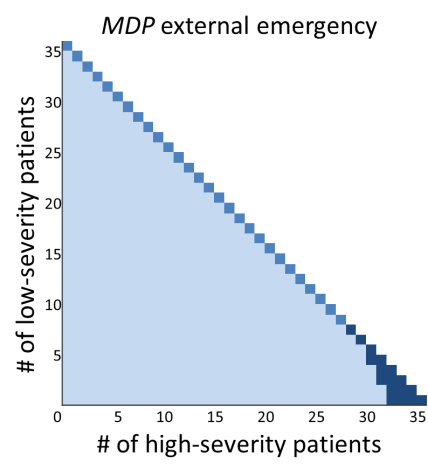 |
| Case 9 (weight medical costs: 0.9) | 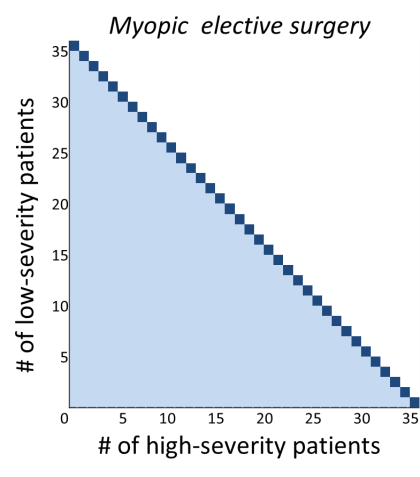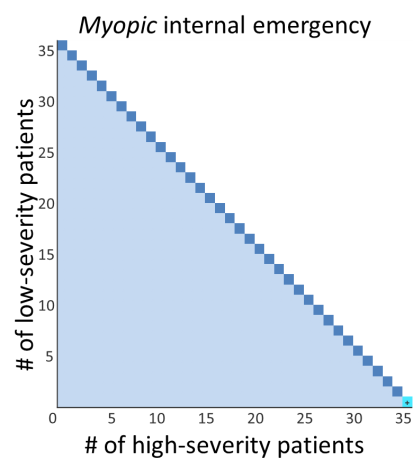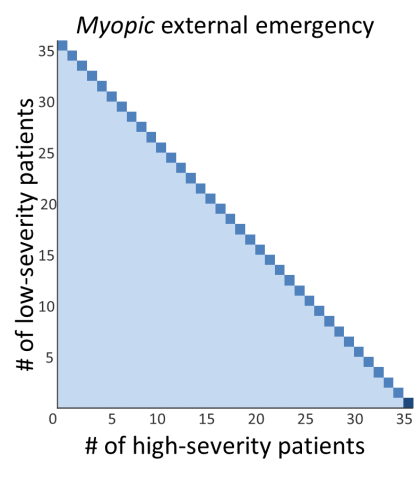 |
|  | 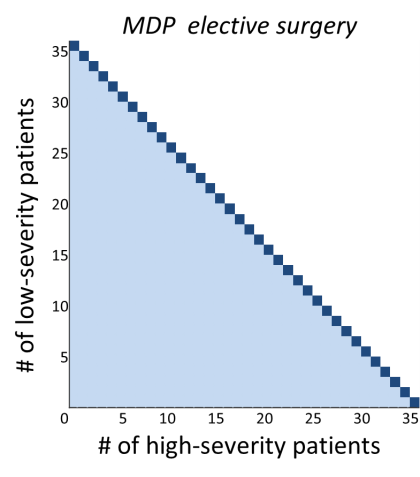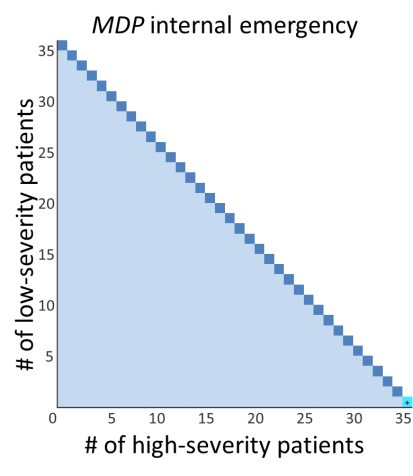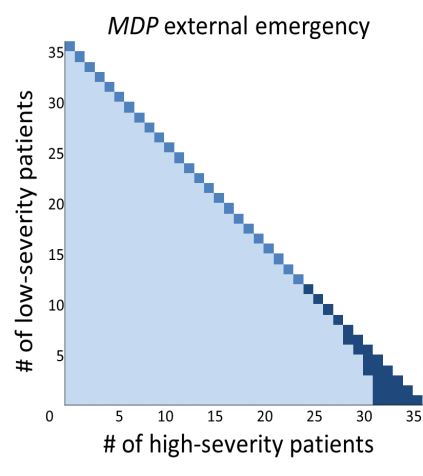 |

| Case 10 (weight medical costs: 1.0) |   |
| --- | --- |
|  | 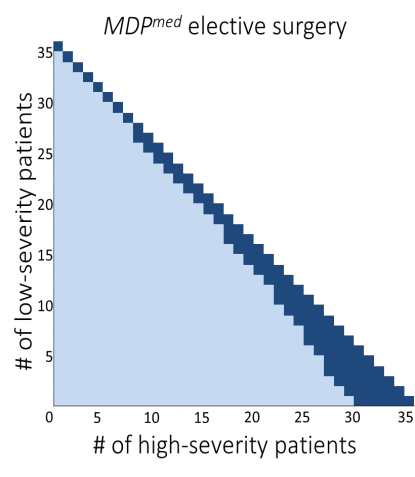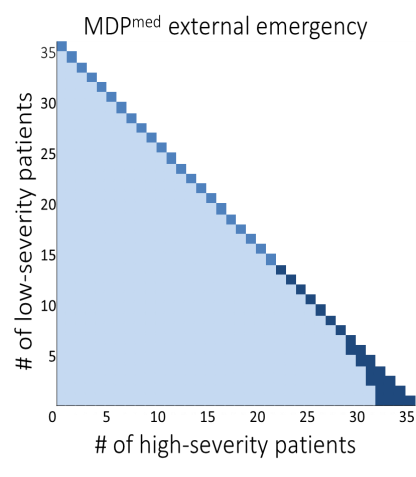 |

Appendix E: Sensitivity Analysis

E.1 Sensitivity analysis I: Variation of parameters

In the following, we perform a sensitivity analysis to check whether the superiority of the MDP approach is robust against variations of the problem parameters. To do so, we use the medical perspective and decrease and increase all cost parameters by 50%. To limit the number of settings, and to concentrate on large variations, we don’t include the base case settings in the study. Combining the two possible values for each of the five cost parameters in a full factorial design yields the 32 test cases given in Table A.3. The cases are described using a sequence of five “+” and “-” (column two) that denote the parameters increased and decreased. The first three symbols indicate the rejection costs for elective surgery, internal emergency, and external emergency, while the remaining two symbols indicate the early discharging costs for low- and high-severity patients. For example, case 3 is denoted by “(- - -; + -)” because the costs of rejecting type 1, 2, and 3 patients (elective surgery, internal emergency, and external emergency, respectively) are decreased, the cost of early discharging a low-severity patient are increased, and the costs of early discharging a high-severity patient are decreased. For each test case, we determined the MDP and myopic policy, and calculate the improvement of MDP’s performance in comparison to the myopic policy applying simulation analysis. The average improvement was 27%, with improvements in the test cases ranging from just under 2% (case 21, (+ - +; - -)), up to almost 50% (e.g., case 15, (- + +; + -)). The standard deviation was 14%, and in only five cases (case 21, 22, 29, 30), we observed improvements below 10% (Table A.3, columns three to five).

| Cases | | | | | | Costs (Correct Estimation)  (Appendix E.1) | | | Costs  (Base Case is true) | | | Relative cost increase due to estimation error | |
| --- | --- | --- | --- | --- | --- | --- | --- | --- | --- | --- | --- | --- | --- |
|  |  |  |  |  |  |  |  |  | (Appendix E.2) | | | | |
| ID | Cost Settings | | | | | MDP | Myopic | Improvement | MDP | Myopic | Improvement | MDP | Myopic |
| 1 | - | - | - | - | - | 964 | 1,221 | 21.04% | 1,929 | 2,443 | 21.04% | -0.11% | -0.41% |
| 2 | - | - | - | - | + | 1,003 | 1,478 | 32.14% | 2,006 | 2,956 | 32.14% | 3.88% | 20.51% |
| 3 | - | - | - | + | - | 1,166 | 2,126 | 45.17% | 2,198 | 2,117 | -3.85% | 13.84% | -13.71% |
| 4 | - | - | - | + | + | 1,255 | 2,204 | 43.07% | 2,030 | 2,276 | 10.81% | 5.12% | -7.22% |
| 5 | - | - | + | - | - | 1,142 | 1,266 | 9.79% | 2,284 | 2,444 | 6.55% | 18.28% | -0.37% |
| 6 | - | - | + | - | + | 1,388 | 1,571 | 11.65% | 2,502 | 2,999 | 16.59% | 29.57% | 22.28% |
| 7 | - | - | + | + | - | 1,520 | 2,902 | 47.63% | 3,023 | 2,426 | -24.63% | 56.57% | -1.11% |
| 8 | - | - | + | + | + | 2,168 | 3,175 | 31.72% | 2,083 | 2,978 | 30.06% | 7.88% | 21.42% |
| 9 | - | + | - | - | - | 969 | 1,214 | 20.23% | 1,937 | 2,428 | 20.23% | 0.31% | -1.00% |
| 10 | - | + | - | - | + | 1,017 | 1,592 | 36.12% | 1,925 | 2,434 | 20.90% | -0.29% | -0.76% |
| 11 | - | + | - | + | - | 1,164 | 2,129 | 45.33% | 2,195 | 2,120 | -3.52% | 13.66% | -13.57% |
| 12 | - | + | - | + | + | 1,255 | 2,228 | 43.68% | 1,981 | 2,115 | 6.32% | 2.60% | -13.79% |
| 13 | - | + | + | - | - | 1,137 | 1,273 | 10.70% | 2,273 | 2,456 | 7.43% | 17.74% | 0.12% |
| 14 | - | + | + | - | + | 1,437 | 1,641 | 12.43% | 2,111 | 2,443 | 13.59% | 9.32% | -0.41% |
| 15 | - | + | + | + | - | 1,519 | 2,920 | 47.99% | 3,020 | 2,443 | -23.63% | 56.39% | -0.42% |
| 16 | - | + | + | + | + | 2,179 | 3,305 | 34.08% | 1,959 | 2,447 | 19.94% | 1.44% | -0.26% |
| 17 | + | - | - | - | - | 1,145 | 1,426 | 19.75% | 2,289 | 2,808 | 18.47% | 18.54% | 14.46% |
| 18 | + | - | - | - | + | 1,165 | 1,703 | 31.59% | 2,323 | 3,334 | 30.32% | 20.31% | 35.92% |
| 19 | + | - | - | + | - | 1,458 | 2,432 | 40.06% | 2,448 | 2,110 | -16.00% | 26.78% | -13.97% |
| 20 | + | - | - | + | + | 1,635 | 2,518 | 35.05% | 2,060 | 2,277 | 9.50% | 6.70% | -7.19% |
| 21 | + | - | + | - | - | 1,434 | 1,461 | 1.81% | 2,869 | 2,789 | -2.85% | 48.57% | 13.71% |
| 22 | + | - | + | - | + | 1,710 | 1,769 | 3.36% | 3,081 | 3,323 | 7.29% | 59.54% | 35.47% |
| 23 | + | - | + | + | - | 2,047 | 3,285 | 37.70% | 3,235 | 2,443 | -32.44% | 67.55% | -0.41% |
| 24 | + | - | + | + | + | 2,908 | 3,543 | 17.92% | 2,071 | 2,983 | 30.57% | 7.26% | 21.61% |
| 25 | + | + | - | - | - | 1,152 | 1,415 | 18.53% | 2,305 | 2,785 | 17.24% | 19.35% | 13.53% |
| 26 | + | + | - | - | + | 1,179 | 1,800 | 34.48% | 2,281 | 2,792 | 18.29% | 18.15% | 13.83% |
| 27 | + | + | - | + | - | 1,458 | 2,490 | 41.44% | 2,448 | 2,119 | -15.53% | 26.78% | -13.62% |
| 28 | + | + | - | + | + | 1,630 | 2,541 | 35.85% | 2,021 | 2,114 | 4.38% | 4.66% | -13.83% |
| 29 | + | + | + | - | - | 1,429 | 1,462 | 2.30% | 2,858 | 2,791 | -2.39% | 47.99% | 13.78% |
| 30 | + | + | + | - | + | 1,753 | 1,831 | 4.27% | 2,663 | 2,779 | 4.18% | 37.89% | 13.28% |
| 31 | + | + | + | + | - | 2,047 | 3,281 | 37.63% | 3,235 | 2,440 | -32.58% | 67.54% | -0.53% |
| 32 | + | + | + | + | + | 2,899 | 3,654 | 20.66% | 1,933 | 2,436 | 20.66% | 0.08% | -0.70% |
| Base case | | | | | | 1,931 | 2,453 | 21.28% | 1,931 | 2,453 | 21.28% | 0.00% | 0.00% |

Table A.3 Result of sensitivity analyses 1 and 2

In the following, we describe the cases where the potential of the MDP is particularly high, and those where the additional value from an MDP is negligible. In general, the main difference is that the MDP policies may reserve beds, while the myopic policies do not (ignoring future effects, it is always dominant to admit a patient when free capacities exist). Thus, the MDP potential is relatively low if the optimal policy does not reserve any beds, that is, when rejection costs are high, and early discharge costs are low. The biggest impact seems to result from changing the costs for deferring ambulances (rejecting external emergencies) and for early discharging low-severity patients: In the cases with high costs for rejecting external emergencies and low costs for early discharging low-severity patients (cases 5, 6, 13, 14, 21, 22, 29, 30), the relative improvement of the MDP is on average 7%, while this figure increases to 41% for the cases with low costs for external emergencies and high costs for early discharging low-severity patients (cases 3, 4, 11, 12, 19, 20, 27, 28). Changing the high-cost parameters (rejecting internal emergencies, early discharging high-severity patients) has little impact on the potential of our model.

We conclude that while our model has significant benefits in most of the considered test cases, there are a few cases where the MDP does not reserve beds, and its use does not lead to a considerable improvement compared to a myopic policy. When rejection costs outweigh the early discharge costs (at least for low-severity patients), myopic policies in fact are similar to MDP policies, resulting in basically no difference.

E.2 Sensitivity analysis II: Robustness against cost misspecifications

As we have seen, the medical cost of ICU decisions can be hard to estimate. Thus, an approach that crucially depends on exactly knowing parameter values may perform very poorly in reality if cost parameters are inaccurately estimated or change over time. To investigate this, we assume that the real medical cost parameters still follow our base case. However, the hospital erroneously believes them to be one of the 32 test cases from the previous analysis and follows the MDP policies from the corresponding test case. This policy is then evaluated using the real (i.e., base case) cost parameters and the result is compared to the optimal MDP policy for the base case (Table A.3, columns six to ten). Thus, the policies and the resulting simulations (number of admissions, rejections and early discharges) for each case are the same for Sections E.1 and E.2. However, we use the cost settings in column two of Table A.3 to calculate the costs in Section E.1 (Table A.3, columns three and four), while we use the base cost settings in Section E.2 (Table A.3, columns six and seven).

Over all 32 cases of biased cost settings, the cost bias leads to an increase of costs of 22% compared to the MDP using the correct cost parameters. There are cases with only little deviation, but also some cases with increases of more than 50%. In the following, we are going to identify critical and less critical misinterpretations of costs and the related implications on policies. The critical cases have in common that rejection costs for scheduled surgeries and external emergencies are overestimated, and that early discharge costs for high-severity patients are underestimated. The average additional costs for these cases (cases 21, 23, 29, 31) amount to 58%, while they are only 3% higher for cases where rejection costs for scheduled surgeries and external emergencies are underestimated and early discharge costs for high-severity patients are overestimated (cases 2, 4, 10, 12). The rationale for those critical cases is that the MDP does not reserve enough beds, and high-severity patients are discharged early.

In contrast to MDP policies, a myopic decision maker is more robust against erroneous estimation of costs – the additional costs per scenario are only around 4%. This is not surprising, as the baseline costs for the myopic case were much higher, and since fewer different policies exist, the possibility to differ is lower. Confronted with biased costs, the MDP still outperforms myopic policies by around 7% (average of column 8 in Table A.3). We now discuss the cases where the myopic policy considerably outperforms the MDP: The most relevant criteria are the estimated costs for early discharging high-severity patients – in case of underestimation, the MDP leads to 4% higher costs compared to the myopic policies (all cases with odd indices), while the MDP reduces total cost compared to myopic policies by 17% when these costs are overestimated (all cases with even indices). Especially in combination with overestimation of rejection costs, leading to a relatively high utilization of the ICU (again, in these cases, patients will not be rejected, both considering MDP and myopic policies), the MDP policies discharge high-severity patients early instead of low-severity patients when these costs are underestimated, leading to strong increases of mortality rates.

We conclude that erroneous estimation of cost parameters may indeed lead to dramatic results. The worst impact on medical costs was observed for combinations of overestimation of rejection costs and underestimation of the cost of early discharges, while results are otherwise relatively robust. Please note that when all costs are scaled (e.g. case 1 and case 32), the MDP policy does not change. The small difference in the results between the policy from case 1 or case 32 and the baseline policy (-0.11% and -0.70%) is due to the stochastic nature of the simulations and lies within the error margin.

**Reference**

[1] W. B. Powell, *Approximate Dynamic Programming: Solving the Curses of Dimensionality*, 2nd ed. Hoboken, New Jersey: John Wiley & Sons, Inc, 2011.

[2] J. P. Grimes, P. M. Gregory, H. Noveck, M. S. Butler, and J. L. Carson, “The effects of time-to-surgery on mortality and morbidity in patients following hip fracture,” *Am. J. Med.*, vol. 112 (9), no. 9, pp. 702–709, 2002.

[3] T. Shiga, Z. I. Wajima, and Y. Ohe, “Is operative delay associated with increased mortality of hip fracture patients? Systematic review, meta-analysis, and meta-regression,” *Can. J. Anesth.*, vol. 55 (3), pp. 146–54, 2008.

[4] A. M. Nyholm, K. Gromov, H. Palm, M. Brix, T. Kallemose, and A. Troelsen, “Time to surgery is associated with thirty-day and ninety-day mortality after proximal femoral fracture: a retrospective observational study on prospectively collected data from the Danish fracture database collaborators,” *J. Bone Jt. Surg.*, vol. 97 (16), no. 16, pp. 1333–1119, 2015.

[5] A. Sabra, F. Daayf, and S. Renault, “Differential physiological and biochemical responses of three Echinacea species to salinity stress,” *Sci. Hortic. (Amsterdam).*, vol. 135, pp. 23–31, 2012.

[6] W. Yibulayin, S. Abulizi, H. Lv, and W. Sun, “Minimally invasive oesophagectomy versus open esophagectomy for resectable esophageal cancer: a meta-analysis,” *World J. Surg. Oncol.*, vol. 14, no. 1, p. 304, 2016.

[7] S. C. Kim, I. Horowitz, K. K. Young, and T. Buckley, “Flexible bed allocation and performance in the intensive care unit,” *J. Oper. Manag.*, vol. 18 (4), no. 4, pp. 427–443, 2000.

[8] S. C. Kim, I. Horowitz, K. K. Young, and T. A. Buckley, “Analysis of capacity management of the intensive care unit in a hospital,” *Eur. J. Oper. Res.*, vol. 115 (1), no. 1, pp. 36–46, 1999.

[9] W. Checkley, “Mortality and denial of admission to an intensive care unit,” *Am. J. Respir. Crit. Care Med.*, vol. 185 (10), no. 10, pp. 1038–1040, 2012.

[10] G. Iapichino *et al.*, “Reasons for refusal of admission to intensive care and impact on mortality,” *Intensive Care Med.*, vol. 36 (10), no. 10, pp. 1772–1779, 2010.

[11] D. B. Chalfin, S. Trzeciak, A. Likourezos, B. M. Baumann, and R. P. Dellinger, “Impact of delayed transfer of critically ill patients from the emergency department to the intensive care unit,” *Crit. Care Med.*, vol. 35 (6), no. 6, pp. 1477–1483, 2007.

[12] A. J. Singer, H. C. Thode Jr, P. Viccellio, and J. M. Pines, “The association between length of emergency department boarding and mortality,” *Acad. Emerg. Med.*, vol. 18 (12), no. 12, pp. 1324–1329, 2011.

[13] C. A. Chrusch, K. P. Olafson, P. M. McMillan, D. E. Roberts, and P. R. Gray, “High occupancy increases the risk of early death or readmission after transfer from intensive care,” *Crit. Care Med.*, vol. 37 (10), no. 10, pp. 2753–2758, 2009.

[14] L. Smith, C. M. Orts, I. O’neil, A. M. Batchelor, A. D. Gascoigne, and S. V. Baudouin, “TISS and mortality after discharge from intensive care,” *Intensive Care Med.*, vol. 25 (10), no. 10, pp. 1061–1065, 1999.

[15] K. Daly, R. Beale, and R. W. S. Chang, “Reduction in mortality after inappropriate early discharge from intensive care unit: Logistic regression triage model,” *Br. Med. J.*, vol. 322, no. 7279, p. 1274, 2001.

[16] C. W. Chan, V. F. Farias, N. Bambos, and G. J. Escobar, “Optimizing intensive care unit discharge decisions with patient readmissions,” *Oper. Res.*, vol. 60 (6), no. 6, pp. 1323–1341, 2012.

[17] InEK GmbH, “G-DRG-System 2017,” *http://www.g-drg.de/G-DRG-System_2017*, 2017. .
